# Individual identity information persists in learned calls of introduced parrot populations

**DOI:** 10.1371/journal.pcbi.1011231

**Published:** 2023-07-27

**Authors:** Grace Smith-Vidaurre, Valeria Pérez-Marrufo, Elizabeth A. Hobson, Alejandro Salinas-Melgoza, Timothy F. Wright

**Affiliations:** 1 Department of Biology, New Mexico State University, Las Cruces, New Mexico, United States of America; 2 Laboratory of Neurogenetics of Language, Rockefeller University, New York, New York, United States of America; 3 Rockefeller University Field Research Center, Millbrook, New York, United States of America; 4 Department of Biological Sciences, University of Cincinnati, Cincinnati, Ohio, United States of America; 5 Department of Biology, Syracuse University, Syracuse, New York, United States of America; 6 Facultad de Biología, Universidad Michoacana de San Nicolás de Hidalgo, Morelia, Michoacán, Mexico; University of Antwerp, BELGIUM

## Abstract

Animals can actively encode different types of identity information in learned communication signals, such as group membership or individual identity. The social environments in which animals interact may favor different types of information, but whether identity information conveyed in learned signals is robust or responsive to social disruption over short evolutionary timescales is not well understood. We inferred the type of identity information that was most salient in vocal signals by combining computational tools, including supervised machine learning, with a conceptual framework of “hierarchical mapping”, or patterns of relative acoustic convergence across social scales. We used populations of a vocal learning species as a natural experiment to test whether the type of identity information emphasized in learned vocalizations changed in populations that experienced the social disruption of introduction into new parts of the world. We compared the social scales with the most salient identity information among native and introduced range monk parakeet (*Myiopsitta monachus*) calls recorded in Uruguay and the United States, respectively. We also evaluated whether the identity information emphasized in introduced range calls changed over time. To place our findings in an evolutionary context, we compared our results with another parrot species that exhibits well-established and distinctive regional vocal dialects that are consistent with signaling group identity. We found that both native and introduced range monk parakeet calls displayed the strongest convergence at the individual scale and minimal convergence within sites. We did not identify changes in the strength of acoustic convergence within sites over time in the introduced range calls. These results indicate that the individual identity information in learned vocalizations did not change over short evolutionary timescales in populations that experienced the social disruption of introduction. Our findings point to exciting new research directions about the robustness or responsiveness of communication systems over different evolutionary timescales.

## Author summary

In some avian and mammalian lineages, vocal communication partially depends on social learning. Learned vocalizations may carry information important to communicate to others, including individual identity or group membership. The information encoded in learned vocalizations may change under different social conditions, such as the number of individuals available for social interactions. We used populations of monk parakeets introduced to the United States of America as a natural experiment of social disruption. We tested the ideas that the type of identity information encoded in learned vocalizations could either remain the same or change in introduced populations compared to native range populations in Uruguay. Using computational approaches, we quantified patterns of acoustic variation linked to identity information in learned vocalizations of native and introduced range populations. We found that individual identity information was more pronounced than group membership in learned vocalizations in each of the native and introduced ranges. The type of identity information important for monk parakeets to communicate appears to have remained the same despite social disruption that occurred over the last 50 years. While socially learned traits are considered very flexible, our findings suggest that the type of identity information encoded in learned vocalizations can be robust to population disruption over cultural timescales.

## 1. Introduction

Animals can use communication signals to transmit social information, including group membership, individual identity, social status, sex, or other social characteristics [1,2]⁠. The types of identity information that animals encode in signals may be an outcome of differences in the social environment within or among species. Different types of information may be more or less important for animals to communicate in social environments that can change over ecological or evolutionary timescales [3–6]⁠.

Vocalizations are well-studied communication signals that can contain identity information. For example, voice cues arising from vocal tract filtering can provide receivers with information about individual identity [7–9]. However, individuals can also use social learning to modify identity information, such as vocal learning species that can encode both group-level and individual identity information in learned vocalizations in a stable manner. When individuals imitate vocalizations of their social companions, the resulting group-level acoustic convergence can be used to recognize group members [10–12]. Learned vocalizations with group identity information, such as vocal dialects, have been reported in several vocal learning taxa, including cetaceans [13–17], bats [18], songbirds [19–21], and parrots [22,23]. Individuals can also communicate individual identity information by developing distinctive vocalizations that differentiate them from other individuals. For instance, bottlenose dolphins (*Tursiops truncatus*) and green-rumped parrotlets (*Forpus passerinus*) can use vocal learning to produce distinctive individual signatures used for individual vocal recognition [24–27].

These findings from the same or closely related taxa suggest that changes in the social environment could influence the identity information that animals encode in learned vocalizations. For instance, living in large social groups or interacting repeatedly with different individuals may favor signaling individual identity information, due to either the pressure of providing sufficient information for receivers to discriminate among unique individuals [28], or the relative benefits and costs associated with maintaining many different social relationships [29]. However, the degree to which identity information encoded in learned communication signals dynamically responds to changes in social conditions over short evolutionary timescales is not well understood.

Short-term changes in the social environment can influence variation within or among types of identity information in learned vocalizations, which could reflect novel changes to the identity information used, or switching among historical forms of identity signaling. For instance, captive and wild Puerto Rican Amazon parrots (*Amazona vittata*) exhibit distinct vocal dialects that have arisen over only a few decades, and translocated individuals will switch between dialects to call in the dialect of the local population [22]. In a field experiment with yellow-naped amazons (*Amazona auropalliata*), a juvenile translocated between regional populations also switched to calling in the local vocal dialect [30]. However, regional dialect boundaries in this species remained stable over 11 years [31], despite natural dispersal of individuals across dialect boundaries [32]. In elephant seals (*Mirounga angustirostris*), increasing population size appears associated with a change in the type of identity information encoded in learned vocalizations over short evolutionary timescales. As recovering populations grew in size over 50 years, vocal dialects were replaced by more structurally complex calls that displayed greater individual distinctiveness, which may facilitate male signaling in more crowded social environments [33]. In eusocial naked mole-rats (*Heterocephalus glaber*), individuals learn colony-specific vocal dialects during development. However, the type of identity information emphasized in learned vocalizations appears sensitive to social stability conferred by the presence of a queen. In a colony that lost two queens within a year, individuals’ chirps became less colony-specific and more individually distinctive during two periods of social instability [34]. This particular change in identity information may be linked to physiological mechanisms of reproductive suppression [34], but still provides compelling evidence that the type of identity information encoded in learned vocalizations can be sensitive to changes in social conditions within an individual’s lifetime.

To test whether identity information in vocalizations is robust or responsive to short-term changes in the social environment, we need two critical components: 1) a way to quantify the relative salience of different types of identity information in learned signals and 2) the potential to compare identity information across groups with different social characteristics.

First, new tools are needed to better quantify the salient types of information in vocalizations. Computational approaches like machine learning can be applied within a conceptual framework that links patterns of vocal convergence to identity signaling. Individuals should use vocal learning to converge on vocalizations across different scales of social organization [35], and such vocal convergence should yield “hierarchical mapping” patterns, which are patterns of relative acoustic convergence that vary in a stable manner across social scales [1]. To evaluate hierarchical mapping patterns, we can use machine learning tools to quantify relative acoustic convergence over different social scales, for example, from individuals to flocks or populations inhabiting different geographic regions. From hierarchical mapping patterns, we can use the social scale with the strongest relative acoustic convergence to infer the most salient type of identity information encoded in vocalizations. This conceptual framework assumes that patterns of acoustic convergence reflect identity information encoding that is stable across social contexts, in contrast to the rapid vocal matching exhibited by some vocal learners that should yield varying patterns of acoustic convergence and divergence in real time [36–39].

Second, we can compare hierarchical mapping patterns among groups with variation in population stability to test whether identity information in learned vocalizations is robust or responsive to disruption of the social environment. We can leverage different types of natural experiments for this comparison, including the introduction of species to new parts of the world, which can cause founder effects that influence traits transmitted by genetic inheritance and by social learning in introduced populations [40,41]. Introduction events that expand a species’ range can be an extreme form of social disruption. In particular, when this process occurs through the pet trade, wild individuals are removed from their natural social environments and are placed in captivity for transport, and then can remain in captivity in breeding colonies that sustain the pet trade throughout the remainder of their lives. These original individuals or their captive-bred descendants can also establish new populations after escaping or being released from captivity [42–44]. New populations established outside of the native range after this form of social disruption should be small shortly after establishment. However, if population growth leads to increased population size after establishment [42], then social environments that are similar to native range populations could gradually re-establish in the introduced range. Alternatively, the effects of social disruption could persist over generations and influence learned vocal outcomes, since vocal learning is a social process. For example, there could be fewer overall numbers of individuals available for social interactions in introduced populations, which could alter the cognitive costs of social recognition for receivers [12,29], and in turn, alter the type of identity information that signalers convey in learned vocalizations compared to the native range.

In this study, we focused on native and introduced range populations of monk parakeets (*Myiopsitta monachus*) to test how social disruption that occurred generations ago, over the course of the past 50 years, could cause changes in the type of identity information encoded in learned calls. Parrots are suitable for this research because they can use social learning to both acquire and modify “contact” calls, which individuals are thought to use to maintain contact with their social companions while flying and foraging [45]. Monk parakeets are particularly useful because they have established new populations worldwide through the pet trade since the late 1960s, enabling comparisons between native range populations and multiple introduced range populations. The independently established introduced range populations share a common origin, with the majority of these populations stemming from native range populations in Uruguay and the surrounding region of northern Argentina [46–49]. In addition, we know more about monk parakeets’ social system than most parrot species. While social relationships among pairs are important, experiments with captive social groups indicate that this species is capable of hierarchical social organization, which could extend to wild populations [50–53]. Finally, recent work has contributed to growing knowledge of this species’ vocal communication system in both the native and introduced ranges [35,54–56].

We used introduced range monk parakeet populations in the United States (U.S.) as independent replicates of populations established following social disruption. Recent work with monk parakeets supports the idea that the introduction process, including transport out of the native range and housing in long-term captivity, represents a form of extreme social disruption. Under naturalistic conditions, removing even a single individual from an established social group consistently disrupts monk parakeets’ dominance ranks [53]. In the U.S. introduced range, social disruption through the pet trade has occurred over short evolutionary timescales, beginning about 50 years ago. The earliest sightings of monk parakeets in the U.S. were reported in 1969, although populations in some states may have been established in the 1980’s or later [46,48]. In our previous work, we used the term “invasive” to refer to monk parakeet populations outside of the native range [35,56]. We now use the more inclusive term “introduced” to refer to these populations [57].

We used contact call recordings to infer which type of identity information was most salient in learned monk parakeet vocal signals. We used this approach on both native and introduced range contact calls to test whether the type of identity information was the same or differed between the native and introduced ranges. Previous work demonstrated that the strongest acoustic convergence in monk parakeet contact calls occurs at the individual scale for native range populations in Uruguay (e.g. strong individual signatures) [35]. In addition, acoustic structures that encoded individual vocal signatures in contact calls of introduced range populations in the U.S. were simpler compared to native range contact calls in Uruguay, which may be associated with signaling and learning in smaller local populations compared to the native range [56]. This work in the introduced range did not assess the relative strength of information encoding at the individual level compared to the group level in contact calls. Whether simpler individual vocal signatures reflect an overall change in the type of identity information encoded in contact calls after population disruption remains unknown, and requires a combined approach to assess convergence at both of the individual and group levels in the native and introduced ranges. We expected that if introduced populations had recovered following social disruption, then the type of identity information in introduced range contact calls would not change, such that both native and introduced populations would exhibit the strongest acoustic convergence at the individual scale. However, if the introduction process was sufficiently disruptive, then we expected that contemporary introduced range parakeets would diverge from the type of identity information used in the native range, and would instead display stronger acoustic convergence at a higher social scale. We placed our results in the context of longer timescales by comparing against another parrot species with strong contact call convergence at higher social scales and distinctive vocal dialects. Our integration of quantitative approaches with a conceptual framework of hierarchical mapping patterns can be used to evaluate stable identity information encoding in learned communication signals more broadly across taxa. Together, our rigorous computational and comparative approaches provide new insight into how identity information in learned vocal signals can be robust to social disruption over ecological timescales, and can differ between species representing longer evolutionary timescales.

## 2. Methods

### 2.1 Ethics statement

This research was conducted under an approved Institutional Animal Care and Use protocol (IACUC no. 2017–006, New Mexico State University, USA) and an animal care and use protocol approved by la Comisión de Ética en el Uso de Animales (CEUA no. 240011-002512-17, la Universidad de la República, Uruguay).

### 2.2 Recording contact calls

We recorded contact calls from native range monk parakeets in 2017 at 37 sites across 7 departments in Uruguay in our previous work [35]. Our introduced range dataset included contact calls recorded at 26 sites across 5 states in the U.S. in 4 different sampling years: 2004, 2011, 2018, and 2019. In 2004, introduced range contact calls were recorded in Connecticut, Florida, Louisiana, and Texas (calls were provided by the authors of [58]). We recorded parakeets in Texas and Louisiana in 2011, Arizona in 2018, and Texas again in 2019. For our temporal analyses below, we relied on contact calls that we recorded in Texas in 2004, 2011, and 2019 (3 sampling years), and contact calls recorded in Louisiana in 2004 and 2011 (2 sampling years, see S1 Appendix section 1).

Recording sessions in 2004 used Marantz PMD670 or PMD690 recorders with Sennheiser ME67K6 shotgun microphones, and these recordings were digitized at 48000 Hz and 16 bit depth [58]. In all other recording sessions we used Marantz PMD661 MKII and PMD660 solid state recorders, Sennheiser ME67 long shotgun microphones and foam windscreens, and we digitized our recordings at 44100 Hz sampling rate and 16 bit depth [35,56]. All recorded individuals were unmarked, with the exception of a few marked individuals in the native range [35].

### 2.3 Pre-processing contact calls

We manually selected contact calls from our field recordings. For our introduced range recording sessions in later years, we selected contact calls using Raven version 1.4 [59], consistent with native range contact call selection in [35]. The previously published introduced range contact calls from 2004 were provided as clips of original recordings [58]. We performed pre-processing for all introduced range contact calls, including the 2004 clips, with the warbleR package [60] to implement the same quality control pipeline we had previously used for native range contact calls (S1 Appendix section 1, [35,56]). Our quality control criteria included contact calls with signal to noise ratios of 7 or higher (e.g. calls that were at least 7 times louder than background noise) that also did not display loud signals or other background noise that overlapped with contact call structure. We performed the majority of our pre-processing and downstream analyses in the R software environment [61], including the tidyverse [62].

### 2.4 Social scales represented in our contact call datasets

We obtained contact calls at two different social scales for the purposes of this study: the individual scale, and a group scale that represented a higher level of social organization. To assess contact call convergence at the individual scale, we repeatedly sampled known individuals to obtain multiple exemplar contact calls produced by the same individual. This individual-level dataset included 229 total contact calls from 8 native range birds (3 marked, 5 unmarked) recorded at 3 different sites in 2017, and 9 introduced range birds (all unmarked) recorded at 7 different sites in either 2004, 2011, or 2019 (see Table A5 in [56]). Each individual was recorded at one site only, and because the birds we recorded were generally unmarked, we recorded repeat contact calls from particular individuals while the calling bird was producing multiple contact calls within a short period of time (e.g. a few minutes [35]). After pre-processing contact calls, our individual scale dataset included a median of 10 (range: 4–25) contact calls for the native range individuals and a median of 12 (range: 5–28) contact calls for the introduced range individuals. Our individual scale dataset provided us with sufficient sampling depth per individual to assess acoustic convergence at the individual scale. We used this contact call dataset to represent individual vocal signatures over a short sampling period for each repeatedly sampled individual. In previous work with this same dataset, we identified individual vocal signatures encoded in frequency modulation patterns [56], which are widely considered to be acoustic structures that animals can modify by learning to create individually distinctive signals [24,26,63,64]. While individuals’ physiological states could influence subtle patterns of variation in learned vocalizations [65], studies with other vocal learning taxa, such as bottlenose dolphins, have also identified individual vocal signatures encoded in the frequency contours of learned vocalizations recorded over short timescales [27,37].

To address contact call convergence at a group scale, we recorded and compared contact calls across nesting sites. We used nesting sites as groups because parakeets likely interact often with other individuals at the same nesting site. Monk parakeet nesting sites include clusters of single or multi-chambered stick nests that are often built in close proximity [66], and parakeets from nearby clusters of nests engage in social interactions [51], making it difficult to determine the boundaries of independent nesting sites. In this study, we recorded at clusters of nests that were geographically separate (the shortest distance among these nesting sites was 0.15 km), which we refer to hereafter as “sites”. For our site scale dataset, we obtained a single contact call per bird at each site. Because the parakeets usually produced only a single contact call when leaving or returning to their nests, we were limited to sampling a single contact call per unmarked individual at this higher social scale.

**Fig 1:**
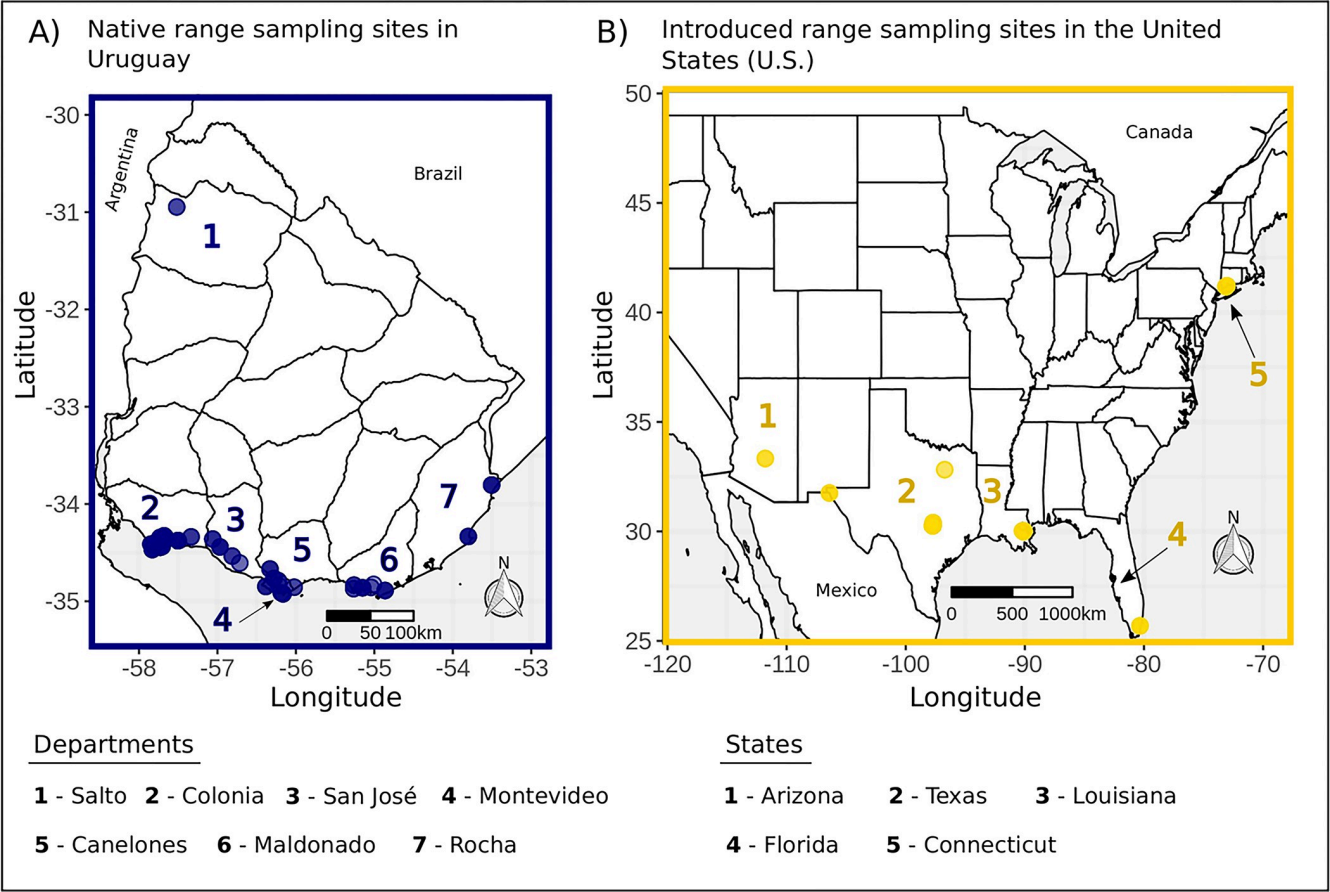
A map of contact call recording sites for native range populations in Uruguay and introduced range populations in the United States (U.S.). We recorded parakeets across A) 7 departments in Uruguay and B) 5 states in the U.S. Our geographic sampling was more contiguous in the native range, which reflected the natural contiguity of populations across the southeastern coast of Uruguay, compared to the more geographically isolated populations in the U.S. introduced range. We used GADM shapefiles for the national and county borders of Uruguay (https://gadm.org/download_country.html). For the U.S., the country and state borders were originally sourced from Natural Earth (https://www.naturalearthdata.com/) and U.S. Census datasets (https://www.census.gov/geographies/mapping-files/time-series/geo/cartographic-boundary.html), respectively.

After pre-processing, our site scale dataset included 1353 total contact calls recorded at 63 sites (37 native range sites and 26 introduced range sites). Some introduced range sites were repeatedly sampled in different sampling years (see Tables A3 and A4 in [56]). This dataset contained a median of 15 (range: 5–53) and 15.5 (range: 5–91) contact calls across the native and introduced range sites, respectively. Since we recorded a single contact call per unique individual at each site, our site scale dataset did not provide sufficient resolution of individual vocal signatures. However, this dataset allowed us to compare patterns of acoustic variation at a higher scale of social organization over broader geographic areas in each range (Fig 1).

To compare hierarchical mapping patterns between the native and introduced ranges, we used 37 native range sites separated by 0.15–513.59 km across 7 departments in Uruguay, and 18 introduced range sites across 5 U.S. states that were separated by 0.74–3502.98 km [35,56]. In our analyses below, we randomly selected a subsample of sites and contact calls per site for calculations of acoustic convergence, and we repeated this process over many resampling iterations, which allowed us to control for non-independence among sites (e.g. sites separated by short geographic distances that may be easily traversed by volant animals). To compare hierarchical mapping patterns over time in the introduced range, we used a subsample of sites in Texas and Louisiana that were recorded in more than one sampling year (see the respective number of sites and geographic distances in S1 Appendix section 1). For our analyses at the site scale, we also generated 3 versions of the site scale dataset to account for the possibility that some contact calls could represent repeated sampling of the same unmarked individual(s) (S1 Appendix section 2). These 3 datasets included the full dataset of contact calls, as well as the full dataset filtered by either clustering with Gaussian mixture models in the mclust R package [67] or visual classification methods with a custom-designed RShiny app [68] to remove contact calls that were likely to represent such repeated individual sampling (S1 Appendix sections 3–7). Following contact call similarity measurements, we performed analyses with these 3 site scale datasets to compare the degree of repeated individual sampling in each of the native and introduced ranges, as well as to assess the robustness of our overall results at this higher social scale. We used separate contact call datasets at the individual and site scales under the assumption that our sampling approach captured stable patterns of acoustic convergence, rather than the rapid vocal matching that some parrots exhibit in real time [36,38,39]. In other words, if individuals were using learning to stably converge on vocalizations at a given social scale, then we expected to find relatively higher convergence at one social scale compared to the other, regardless of the individuals that we sampled at each social scale.

### 2.5 Measuring contact call similarity with spectrographic cross-correlation

We used contact call similarity measurements to quantify hierarchical mapping patterns across different social scales. For instance, if individuals were converging on shared contact calls within sites, then we expected that contact calls compared within the same site would exhibit high similarity measurements, and lower similarity measurements when compared to contact calls from different sites. We measured contact call similarity with spectrographic cross-correlation (SPCC) [69], which has traditionally been used in studies reporting patterns of acoustic variation consistent with social learning of vocalizations in parrots [23,24,30,31,35,36,38,58,70–73]. We performed SPCC with a Hanning window, a window length of 378 samples, and a window overlap of 90 samples for Fourier transformations, as well as Pearson’s correlation method and a bandpass filter of 0.5 to 9kHz [60]. Unless otherwise specified, we used these same parameters for subsequent spectrum-based analyses. We conducted SPCC with all contact calls across the native and introduced ranges.

### 2.6 Measuring contact call similarity with supervised machine learning

We also measured similarity among monk parakeet contact calls using a supervised machine learning approach that identifies biologically relevant patterns of variation in avian acoustic signals [35,74,75]. As in our previous work [35], measuring similarity with a traditional method (SPCC) and a newer method (supervised random forests), allowed us to verify that the hierarchical mapping patterns we identified were not an artifact of using a single similarity method. We built supervised random forests models with 1844 acoustic and image features, including features derived from SPCC and dynamic time warping similarity measurements, standard spectral acoustic measurements, descriptive statistics of Mel-frequency cepstral coefficients, and spectrogram image measurements (S1 Appendix sections 8–9). We used the warbleR and dtw R packages for acoustic measurements [60,76], the software WNDCHRM for image measurements [77], and the MASS and base R packages to extract features [61,78]. We trained random forests models to classify contact calls back to 4 repeatedly sampled individuals in each of the native and introduced ranges with the caret package (156 contact calls and 8 individuals total, S1 Appendix sections 10–11) [79,80]. We built and trained models on known repeatedly sampled individuals because native range monk parakeet contact calls group visibly by individual in a low dimensional trait space (e.g. two-dimensional acoustic space, S1 Fig) [35]. It is important to train classification models on discrete categories or classes [81], as a means of ensuring that classification outcomes reflect biologically relevant variation, rather than issues with how the models were built.

We built our first model with the full set of 1844 acoustic and image features. We built a second model by performing automated feature selection and using the most important features from that analysis (S1 Appendix section 11). Our second model outperformed the first model, so we used the second model with 114 features for final analyses. To predict the similarity of the individual scale contact calls that we used for validation, as well as the site scale contact calls, we ran the remaining individual scale contact calls (73 total contact calls, 4 and 5 repeatedly sampled native and introduced range individuals, respectively) and the 1353 site scale contact calls down the final model. We extracted the resulting proximity matrix as the random forests similarity measurements [35,74,75,82,83]. We performed our random forests analyses with the caret, ranger, Boruta, and edarf R packages [80,84–86]. To validate model performance, we used these similarity measurements to cluster the validation contact calls with Gaussian mixture modeling in the R package mclust [67], which allowed us to determine whether the random forests model identified biologically relevant patterns of acoustic variation within and among contact calls of new individuals (e.g. individuals that were not present in the training dataset).

After confirming that the final model captured relevant patterns of variation among the individuals that we used to validate model performance, we used random forests similarity measurements to generate low-dimensional acoustic space visualizations. Since we had used the individual scale contact calls to train and validate the random forests model that we used to predict contact call similarity, we did not use random forests similarity measurements to perform quantitative analyses of acoustic convergence at the individual scale. Instead, we used the training classification performance of our final random forests model, and the clustering performance during validation with random forests similarity, to support our individual scale analyses with SPCC similarity. Using two similarity methods to quantify acoustic convergence at the site scale allowed us to validate that our results at this social scale reflected biologically relevant variation, and did not arise from relying on a single similarity method.

### 2.7 Comparing native and introduced range hierarchical mapping patterns in acoustic space

To assess hierarchical mapping patterns in each of the native and introduced ranges, we compared patterns of acoustic convergence in low-dimensional acoustic space at the individual and site social scales. To generate acoustic space for each similarity method, we optimized non-metric multidimensional scaling (MDS) to reduce the dimensionality of the SPCC and random forests similarity matrices, respectively, with the MASS R package [78] (S1 Appendix section 12). For acoustic space at the individual scale, we used random forests similarity obtained during model validation for 4 native range parakeets recorded at 3 sites in the department of Colonia, Uruguay in 2017, and 4 introduced range birds recorded at 3 sites in Austin, Texas, U.S. in 2019. For the site scale, we used both random forests and SPCC similarity measurements for 4 native range sites in the department of Colonia, Uruguay in 2017, and 4 introduced range sites in Austin, Texas, U. S. in 2019. We also filtered the acoustic space MDS coordinates by contact calls in each of the 3 site scale datasets that we used to address repeated sampling of individuals (see section 2.4). Acoustic space can be interpreted on the same axes for each similarity method but not compared between similarity methods (e.g. acoustic space is different between SPCC and random forests analyses). We interpreted contact calls that grouped together in acoustic space by individual or site as structurally similar calls (e.g. high convergence), while calls dispersed in acoustic space were structurally different (e.g. low convergence). We compared hierarchical mapping patterns between the native and introduced ranges by comparing the relative patterns of overlap in acoustic space among individuals or sites.

### 2.8 Using Earth Mover’s Distance to compare hierarchical mapping patterns between ranges

Mantel tests have traditionally been used to correlate matrices of acoustic similarity with matrices of binary categorical identity (e.g. individual or group identity) over many permutations, in order to address whether vocalizations compared within categories are more similar than vocalizations among categories (S1 Appendix sections 15–16), while also controlling for non-independent data in pairwise symmetric matrices [23,35]. Due to recent criticism of using Mantel tests to quantify acoustic convergence [54], we instead used Earth Mover’s Distance, or the minimum amount of work needed to convert one distribution into another [87] to estimate the strength of acoustic convergence across social scales. Earth Mover’s Distance provides a conceptually similar approach to Mantel tests that can be used to quantify and compare acoustic convergence. We compared hierarchical mapping patterns between the native and introduced range populations by comparing the relative magnitude of Earth Mover’s Distance values at each social scale between ranges.

For this analysis, we obtained similarity values representing comparisons of contact calls within and among categories at each social scale (e.g. comparisons of the same or different individuals at the individual scale). We used the emdist R package [88] to calculate Earth Mover’s Distance as the minimum amount of work needed to convert distributions of the same-category contact call comparisons into distributions of different-category contact call comparisons. We performed these calculations in a single dimension bounded between 0 and 1 (e.g. the minimum and maximum possible similarity values). In these calculations, larger values of Earth Mover’s Distance are equivalent to stronger acoustic convergence. For instance, if stronger convergence occurred at the individual scale, then similarity values for contact calls compared for the same individual should be distributed closer to 1, while similarity values for contact calls compared among individuals should be distributed closer to 0, and it should take more work, or greater Earth Mover’s Distance, to convert one distribution into the other. We calculated Earth Mover’s Distance in a histogram-based approach with a customized resampling routine to generate even sample sizes for calculations across social scales. Our resampling routine also allowed us to control for variation in same-site membership at the individual scale, as well as possible non-independence among sites at the site scale (S1 Appendix section 13).

### 2.9 Evaluating hierarchical mapping patterns over time in the introduced range

We compared the relative magnitudes of Earth Mover’s Distance calculations over time in two U.S. cities to determine whether the strength of acoustic convergence at the site scale changed over time in the introduced range. For these analyses, we used introduced range populations that we had repeatedly recorded in Austin, Texas and New Orleans, Louisiana. We calculated Earth Mover’s Distance with the emdist package [88] with our customized resampling routine for each year that we had sampled contact calls in each city, because we did not always sample the same sites in each year. For Austin, we obtained Earth Mover’s Distance using different sites recorded in each of 3 sampling years: 3 sites in 2004, 5 sites in 2011, and 6 sites in 2019. For New Orleans, we calculated Earth Mover’s Distance using different sites sampled in 2 years: 3 sites in 2004 and 2 sites in 2011. We obtained Earth Mover’s Distance with random forests and SPCC similarity measurements, as well as each of the 3 site scale datasets (the number of sites used in each city and year was smaller for the datasets filtered after clustering and visual classification, S1 Appendix section 13). These analyses were similar to those that we performed above to compare hierarchical mapping patterns between ranges (section 2.8, S1 Appendix section 13). We also performed Mantel test results over time in these introduced range cities (S1 Appendix section 17). Finally, we addressed the possibility of population recovery since introduction by using the auk R package [89]⁠ to evaluate population trends from eBird checklists in each city over our sampling years (S1 Appendix section 14) [90].

### 2.10 Comparing hierarchical mapping patterns with another parrot species

We placed our results in a broader context by quantifying and directly comparing hierarchical mapping patterns of native and introduced range monk parakeets with the yellow-naped amazon, a species well-known for having regional group identity information in their contact calls. These amazon parrots imitate the contact calls of conspecifics and exhibit distinctive regional vocal dialects that are audibly perceptible to humans [23]. Such vocal sharing may facilitate recognizing familiar group members [12,23]. Regional dialects in yellow-naped amazon contact calls have provided a baseline for identifying strong acoustic convergence within social groups for other vocal learning species [58,70,72], including monk parakeets [35]⁠. Here we used yellow-naped amazon contact calls as a point of reference for strong acoustic convergence that could occur at a higher social scale in introduced range monk parakeet contact calls if group membership information became more important to signal after introduction than individual identity.

For our comparative analyses, we quantified hierarchical mapping patterns over the individual and site social scales for native and introduced range monk parakeets (separately), and over the individual, site, and regional dialect social scales for yellow-naped amazons. For yellow-naped amazons, we used previously published contact calls recorded in Costa Rica in 1994 [23]. We measured contact call similarity for each species using SPCC [60], and selected similarity values for a subsample of individuals or groups at each social scale that represented similar sampling depth and geographic breadth for each range and species (S1 Appendix sections 19–20). We compared hierarchical mapping patterns by assessing patterns of relative overlap among distributions of the subsampled SPCC similarity values within and among categories (e.g. individuals or groups).

We also designed a customized bootstrapping approach to quantify the strength of acoustic convergence at each social scale for native range monk parakeets, invasive range monk parakeets, and yellow-naped amazons that complemented and validated our analyses with Earth Mover’s Distance. In this analysis, we randomly selected 5 SPCC similarity values within the given categories and 5 SPCC similarity values among the given category in each bootstrapping iteration (S1 Appendix section 21). This random sampling was performed with replacement, such that SPCC values within or among categories could be randomly selected more than once in the same iteration. We calculated bootstrapped similarity ratios by dividing similarity values within the given category by similarity values among the given category. We performed bootstrapping over 200 iterations and calculated 1000 total similarity ratios for exemplars of each category (individual or group) at each social scale for native range parakeets, introduced range parakeets, and yellow-naped amazons. Similarity ratios close to 1 pointed to weaker convergence. We used similarity ratios increasingly greater than 1 as evidence of stronger convergence (e.g. contact calls were more similar within categories than among categories).

## 3. Results

### 3.1 Strong individual signatures in native and introduced range contact calls

**Fig 2:**
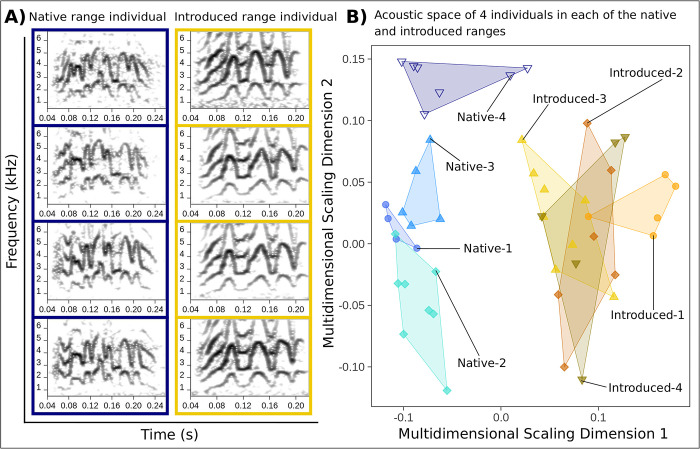
Native and introduced range monk parakeets displayed strong individual vocal signatures. In A) we show a lexicon with 4 contact calls for one repeatedly sampled bird in each of the native and introduced ranges. In B), random forests acoustic space is shown for 4 native range and 4 introduced range individuals. Each point represents a different contact call per individual, and individual identities are encoded by shapes and hues. The convex hull polygons demonstrate the area per individual in acoustic space. The blue palette corresponds to the native range and gold-brown to the introduced range. See Table A in S1 Appendix for decoded individual identities. Individuals generally produced visibly consistent contact calls (A) that were also distinctive from other individuals (B).

We identified strong acoustic convergence at the individual scale in contact calls recorded in both ranges. Contact call lexicons (or collections of spectrograms) for known repeatedly sampled individuals indicated that parakeets in each of the native and introduced ranges consistently produced contact calls that were distinctive from those of other birds (Fig 2A). This result was further supported by the general patterns of low overlap among individuals that we identified in random forests and SPCC acoustic space, although there was higher overlap among introduced range individuals (Figs 2B and S1).

Our supervised machine learning results also pointed to strong acoustic convergence at the individual scale. The final random forests model that we used for prediction displayed high classification accuracy during training. The model classified contact calls back to the individuals that we used for training with 97.44% accuracy (95% CI: 93.57–99.30%). The mean ± SE balanced accuracy of our model’s classification performance per individual (representing the averaged sensitivity and specificity) was similarly high for the 4 native range (99.00% ± 1%) and 4 introduced range training individuals (98.75% ± 0.75%). Finally, our analyses of the strength of acoustic convergence at the individual scale with Earth Mover’s Distance also supported strong individual signatures in native and introduced range contact calls. The Earth Mover’s Distance values that we calculated at the individual scale in each of the native and introduced ranges were of similar magnitude (Native range mean and 95% CI: 0.159 (0.153, 0.164); Introduced range mean and 95% CI: 0.131 (0.125, 0.138), Table B in S1 Appendix). We obtained qualitatively similar results using Mantel tests (S1 Appendix section 16, Table D in S1 Appendix).

### 3.2 Contact call convergence within sites was low

**Fig 3:**
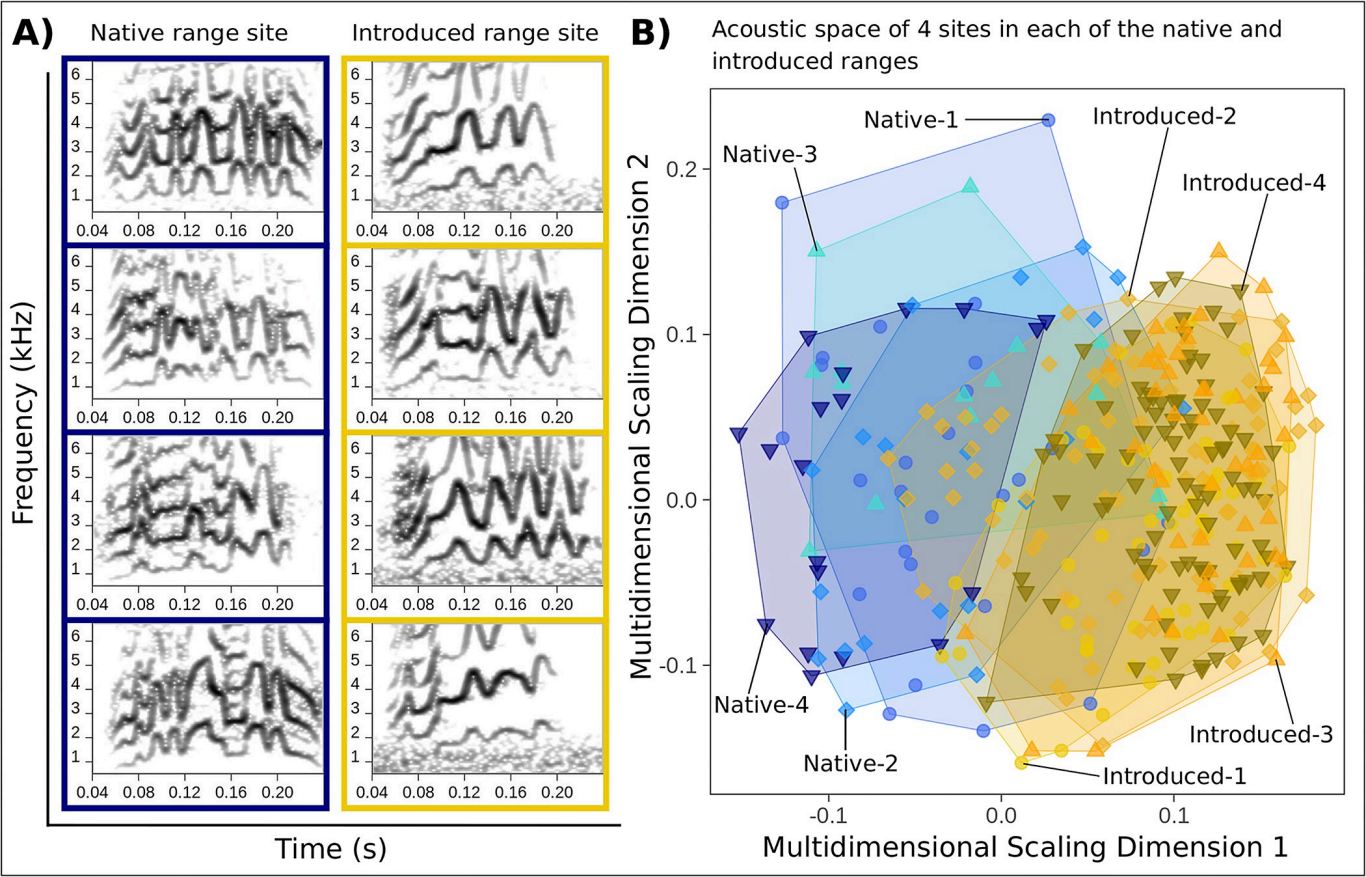
We identified minimal acoustic convergence at the site scale in the native and introduced ranges. In A) we show a lexicon of 4 contact calls each for one native range site and one introduced range site, in which each contact call represents a unique individual. B) is a plot of random forests acoustic space for 4 native range and 4 introduced range sites. The full dataset of contact calls was used per site (see S2 Fig for the other site scale datasets). Across panels, the color palettes, aesthetics, and polygons used are similar to Fig 2, but here encode site identities. See Table A in S1 Appendix for decoded site identities. Contact calls within sites were visibly different (A), and there was low differentiation among sites in acoustic space (B) compared to the individual scale (Fig 2B).

**Fig 4:**
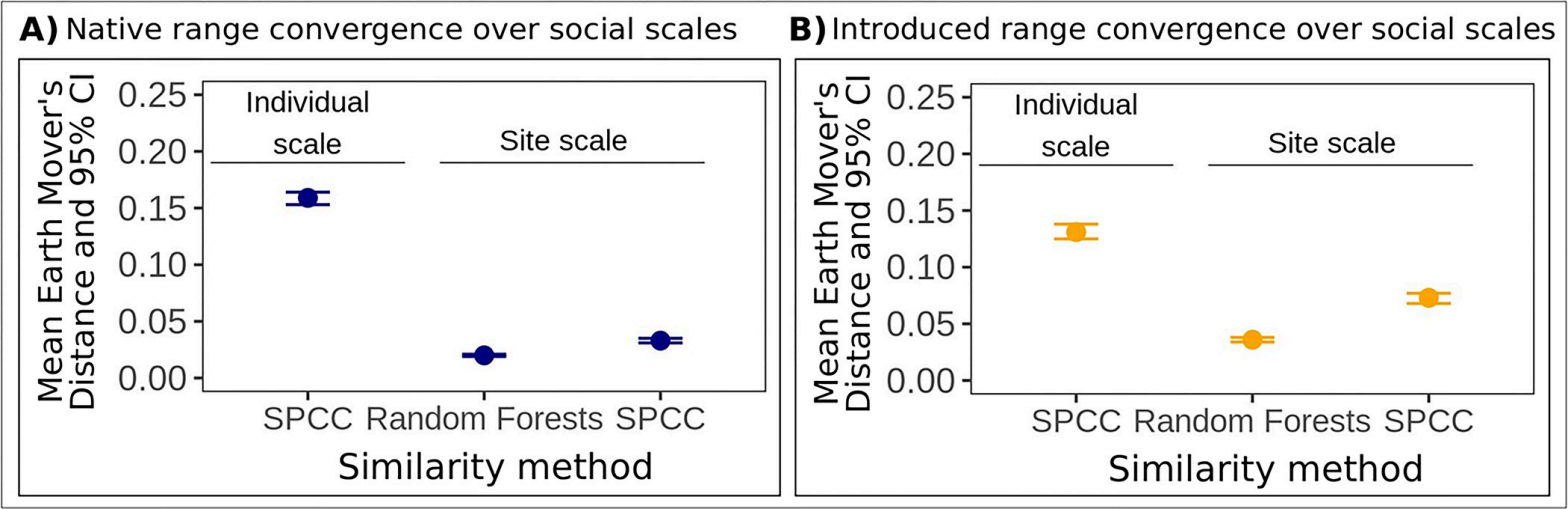
Acoustic convergence was stronger at the individual scale for native and introduced range monk parakeets. We show Earth Mover’s Distance measurements for A) native range monk parakeets, and B) introduced range monk parakeets. In each panel, the symbols and error bars show the mean individual and site scale Earth Mover’s Distance values and 95% confidence intervals calculated with spectrographic cross-correlation (SPCC) or random forests similarity. Higher Earth Mover’s Distance values indicate higher convergence, and we identified higher convergence at the individual scale than at the site scale in both the native and introduced ranges. The site scale values were calculated with the full contact call dataset at this social scale.

We found that individuals at the same site did not produce similar contact calls (Fig 3A). When we assessed hierarchical mapping patterns in acoustic space, we found that contact calls did not group by site identity. Instead, contact calls from the same site were overdispersed, resulting in substantial overlap among different sites in acoustic space generated using random forests similarity (Fig 3B), as well as SPCC similarity (S2 Fig). The low degree of acoustic convergence that we identified at the site scale was supported by Earth Mover’s Distance values that were an order magnitude lower for the site scale compared to the individual scale in each of the native and introduced ranges (Fig 4 and Table B in S1 Appendix). This result held across the complementary SPCC and random forests similarity methods that we used for Earth Mover’s Distance calculations at the site scale (Fig 4).

We compared our Earth Mover’s Distance results across the 3 site scale datasets to determine how keeping or filtering out contact calls of potentially repeatedly sampled individuals affected our results at this social scale. While the Earth Mover’s Distance statistics for the 3 native range site scale datasets were consistently low, values for the introduced range varied more across the site scale datasets. The introduced range Earth Mover’s Distance values for each site scale dataset were uniformly greater than those we obtained for the native range datasets using each similarity method (Table B in S1 Appendix). However, despite this variation that we observed between ranges, and across site scale datasets for the introduced range, all Earth Mover’s Distance values at the site scale remained an order of magnitude lower than the values we calculated at the individual scale in each of the native and introduced ranges (Fig 4 and Table B in S1 Appendix). The highest Earth Mover’s Distance values that we observed at the site scale for the native and introduced ranges occurred with the full dataset of contact calls, in which we did not filter out contact calls attributed to repeatedly sampled unmarked individuals at this social scale (Fig 4 and Table B in S1 Appendix). We obtained similar results using Mantel tests (Table D in S1 Appendix).

### 3.3 Patterns of site scale convergence in the introduced range were consistent over time

**Fig 5:**
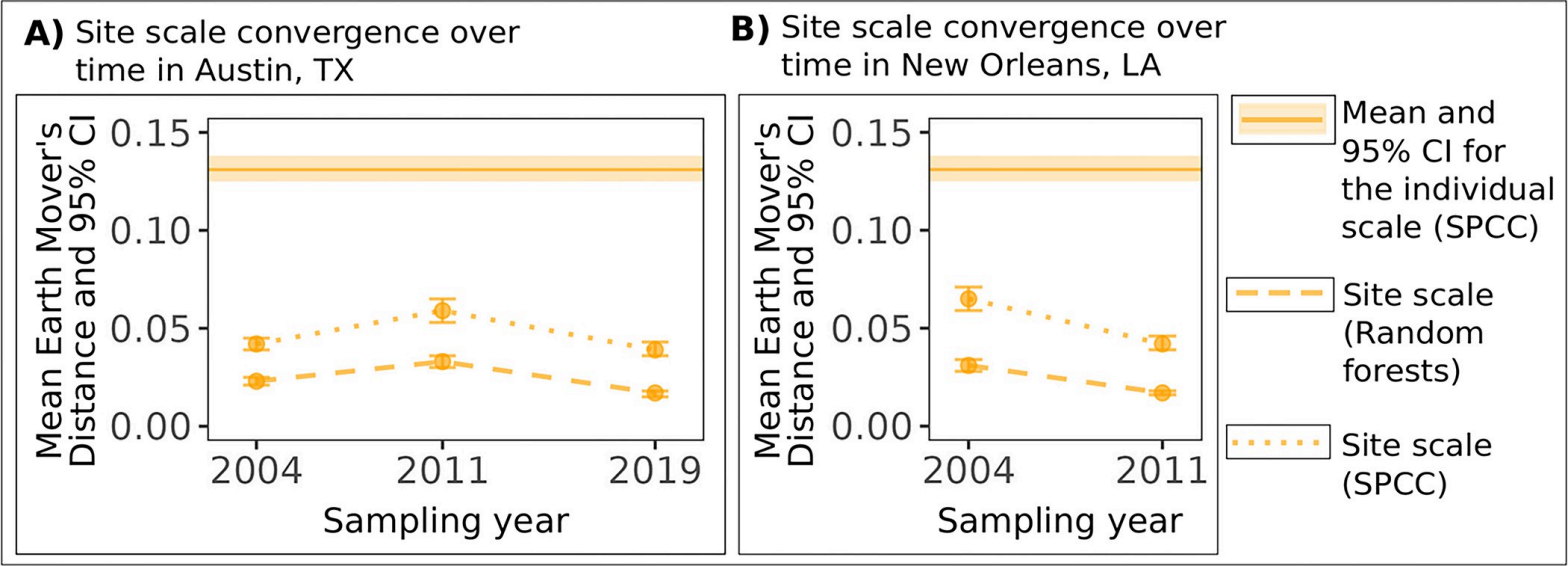
Introduced range acoustic convergence at the site scale remained low in two cities sampled over time. We show Earth Mover’s Distance measurements for A) 3 sampling years in Austin, TX and B) 2 sampling years in New Orleans, LA. The mean Earth Mover’s Distance value calculated for the individual scale with SPCC similarity is shown as a point of reference (a solid horizontal line in each panel). The shading around the individual scale line represents the 95% confidence interval. Lower Earth Mover’s Distance values indicate lower convergence, and site scale convergence over time in each city remained lower than individual scale convergence for the introduced range. In each panel, the symbols and error bars show the mean site scale Earth Mover’s Distance values and 95% confidence intervals calculated with random forests (dashed lines) or spectrographic cross-correlation (SPCC) similarity (dotted lines). The site scale values were calculated with the full contact call dataset at this social scale.

We did not identify clear evidence of temporal change in the strength of site scale acoustic convergence in the introduced range (Fig 5 and Table C in S1 Appendix). In the city of Austin, we identified higher Earth Mover’s Distance values (indicating higher convergence) in 2011 using all 3 site scale datasets for both SPCC and random forests similarity (Table C in S1 Appendix). For the city of New Orleans, we found the highest Earth Mover’s Distance values in 2004 using the full and visual classification datasets and both similarity methods (Table C in S1 Appendix). Despite this variation, the Earth Mover’s Distance values never reached the same magnitude as convergence at the individual scale (Fig 5), but rather remained at the same order of magnitude over time in each city (Table C in S1 Appendix). These Earth Mover’s Distance values that we calculated over time in each city were similar to the site-level calculations that we obtained in our comparison between ranges (Tables B and C in S1 Appendix), and we found similar results using Mantel tests (Table E in S1 Appendix). We used eBird checklists from these cities in an analysis of population trends over time, to address the possibility that population size could have increased since establishment. However, we found that the mean annual frequency of monk parakeets reported in complete checklists in Austin and New Orleans remained low (less than 5% of all species sightings) and was also generally consistent from 2004 to 2020 (S1 Appendix section 14 and S7 Fig).

### 3.4 More repeated sampling of individuals in our introduced range site scale dataset

Using clustering with Gaussian mixture models, and visual classification across multiple observers, we attributed more contact calls in our introduced range site scale datasets to the inadvertent repeated sampling of unmarked individuals compared to our native range site scale datasets. The mean number of repeated individuals that we identified by our clustering and visual classification filtering approaches were only slightly higher for the introduced range than the native range (Table 1). However, we found that the mean number of contact calls attributed to repeated individuals was nearly twofold greater for introduced range sites by each of the clustering and visual classification approaches that we had used to identify repeated sampling of individuals in our site scale datasets (Table 1).

**Table 1:** Assessing the degree of repeated sampling of individuals at the site scale in each of the native and introduced ranges.

Filtering approach	Range	Repeated individuals (mean ± SE)^a^	Contact calls per repeated individual (mean ± SE)^b^
Clustering	Native	3.14 ± 0.37	10.0 ± 1.60
	Introduced	3.23 ± 0.51	18.0 ± 4.75
Visual classification	Native	3.48 ± 0.39	2.83 ± 0.15
	Introduced	3.57 ± 0.54	5.31 ± 0.64

^a,b^These statistics were calculated with 36 native range and 22 introduced range sites, respectively, after removing 4 sites used for multi-observer reliability of visual classification (S1 Appendix section 6).

### 3.5 Distinct hierarchical mapping patterns between monk parakeets and yellow-naped amazons

**Fig 6:**
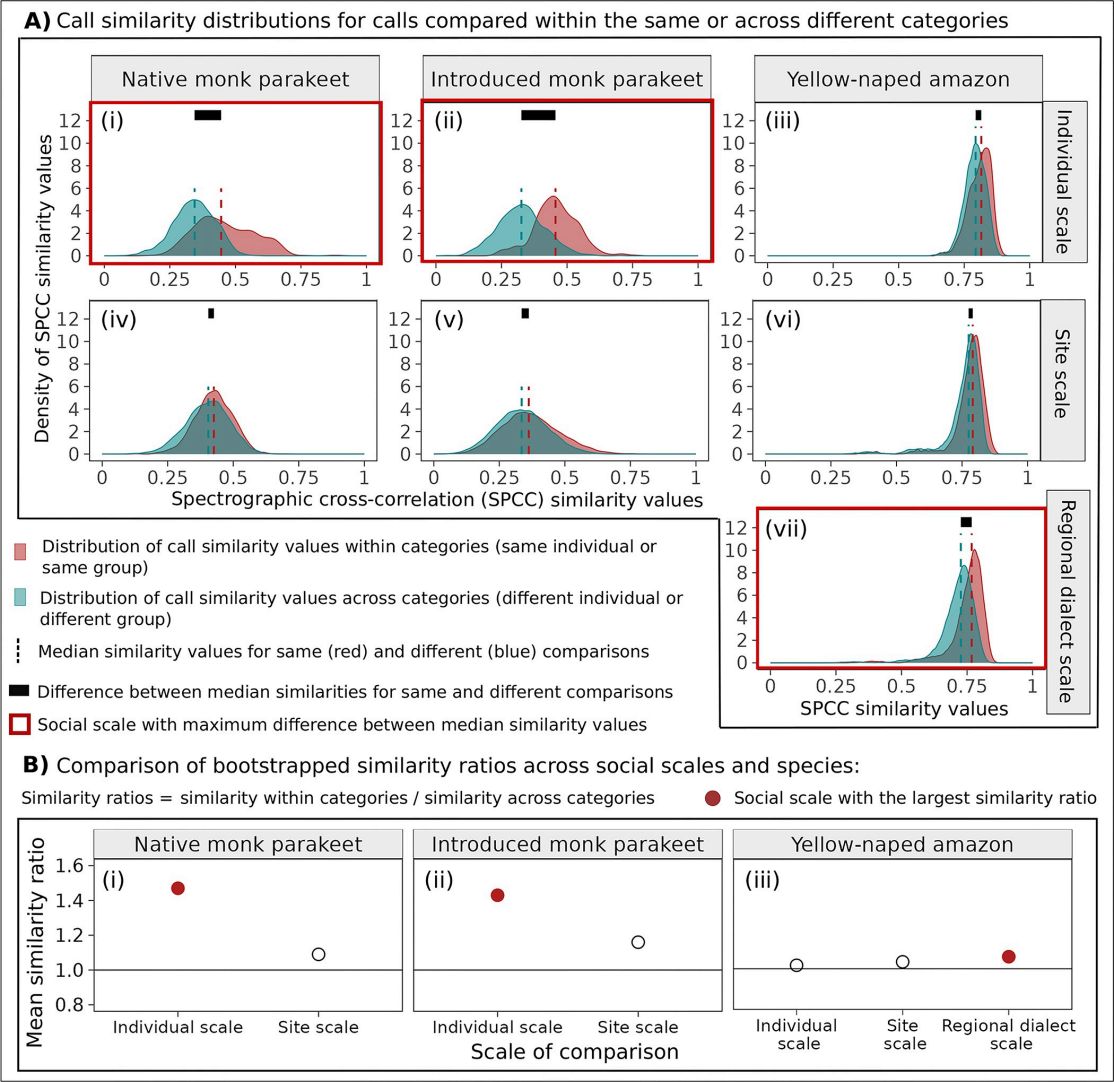
We compared hierarchical mapping patterns among contact calls of native and introduced range monk parakeets as well as yellow-naped amazons. In A) we show density curves for the distributions of spectrographic cross-correlation (SPCC) similarity values that represent comparisons of contact calls within or among categories in red and blue shading, respectively. The dashed lines represent the median similarity values per distribution. In B) we show the mean similarity ratios calculated from bootstrapped SPCC values. The solid line at 1 represents no convergence within a given category. For both native and introduced range monk parakeets, we show site scale results obtained from the full dataset of contact calls. In both A) and B), the social scale at which the strongest convergence occurred is shown in red.

The hierarchical mapping patterns that we identified for both native and introduced range monk parakeet contact calls differed from the hierarchical mapping patterns that we recapitulated in yellow-naped amazon contact calls. Our results from this comparative analysis showed that the individual scale was the social scale with the strongest acoustic convergence in native and introduced range monk parakeet contact calls, while the regional dialect scale displayed the strongest convergence in yellow-naped amazon contact calls. We found that the greatest separation between the median similarity values of the two categories of comparison per social scale (e.g. same or different individual or group) occurred at the individual scale for native and introduced range monk parakeets (Fig 6A, panels i and ii). For yellow-naped amazons, we detected the greatest separation between categories at the regional dialect scale (Fig 6A, panel vii). In addition, the bootstrapped similarity ratios that we used to assess the strength of acoustic convergence were greatest at the individual scale for monk parakeets in each of the native and introduced ranges (Fig 6B, panels i and ii). In contrast, the largest similarity ratio for yellow-naped amazons occurred at the regional dialect scale (Fig 6B, panel iii).

## 4. Discussion

We asked whether the type of identity information that is important to communicate in learned acoustic signals changed in introduced populations established after social disruption that occurred over ecological timescales. We inferred that individual identity remained the most important type of identity information to communicate in learned monk parakeet vocalizations, even in populations established after repeated introductions to new parts of the world. We discuss this new insight into the robustness of identity information encoded in learned communication signals, and point to possible directions for future work over ecological and evolutionary timescales.

### 4.1 Hierarchical mapping patterns were similar between native and introduced range monk parakeet populations

Monk parakeets in native range populations in Uruguay and introduced range populations in the U.S. emphasized individual identity information in learned vocalizations. In each range, the hierarchical mapping patterns that we quantified in contact calls showed the strongest convergence at the individual scale and weaker convergence within sites. These results were robust to the greater degree of repeated individual sampling that we identified in our introduced range site scale dataset (Table 1 and S1 Appendix section 18). In addition, the low convergence that we identified at the site scale in two cities sampled over time, which represented independent introduction events, suggested that these hierarchical mapping patterns were unlikely to have changed in the broader U.S. introduced range over the timespan of this study. We also recapitulated the structural differences previously identified between native and introduced range contact calls that reflected the simplification of individual vocal signatures associated with smaller local populations in the U.S. (see the separation in acoustic space among native and introduced range contact calls in Figs 2B and 3B) [56]. This simplification of individual vocal signatures post-introduction may explain the patterns of greater overlap that we identified among introduced individuals in acoustic space (Fig 2), as well as lower acoustic convergence at the individual scale for the introduced range compared to the native range using Earth Mover’s Distance (Fig 4 and Table B in S1 Appendix). However, despite these differences at the individual scale between ranges, we found that acoustic convergence at the individual scale was consistently an order of magnitude greater than convergence at the site scale in each of the native and introduced ranges. This overall result of stronger convergence at the individual scale and weaker convergence at the site scale in monk parakeet contact calls was supported by the two methods that we used to measure call similarity (SPCC and random forests), as well as the two analytical approaches that we used to quantify acoustic convergence (Earth Mover’s Distance and a customized bootstrapping routine). Using two methods to measure contact call similarity, as well as two methods to quantify acoustic convergence, allowed us to validate the weaker convergence that we identified at the site scale in each of the native and introduced ranges.

Our analyses indicate that individual identity remained the most important type of identity information to communicate to receivers, even in introduced populations. In other words, we inferred that the type of identity information emphasized in learned contact calls was robust to social disruption that occurred over short evolutionary timescales (less than 50 years ago when monk parakeets were introduced to the U.S. [46,48]). Although some features of the social environment changed after introduction, such as the smaller local population sizes that we identified in previous work [56], monk parakeets’ social environments may have been generally robust to introduction or were re-established after initial perturbations. If the individually distinctive contact calls that we identified in the native and introduced ranges are used for individual vocal recognition, then parakeets in each range should be engaging in social interactions that favor signaling individual identity in learned communication signals, which is an idea that can be tested in future work. Our quantitative approaches with vocal signals allowed us to reach this inference without depending on the time- and resource-intensive collection of social data. These findings do not preclude the possibility that social interactions at higher scales of social organization are important in this species. While relationships at the pair level are important for monk parakeets, this species consistently forms social groups with multiple levels of social organization in captive settings [50–53,91].

Signaling individual identity information in learned vocalizations could instead reflect a more fixed aspect of vocal communication systems, such as developmental constraints or genetic encoding of receivers’ perceptual abilities. Future work could also address the stability of individual identity information encoding in learned contact calls across different social contexts, given that some vocal learning species exhibit rapid convergence or divergence that appears conditional on the social context [36–39], and in others, individual vocal signatures [92] or individually-distinctive repertoires of shared contact calls appear to change over time [93].

### 4.2 Comparing our results against a parrot species that exhibits regional vocal dialects

We performed a comparative analysis with yellow-naped amazon contact calls to place our ecological comparison of native and introduced range monk parakeet contact calls in an evolutionary context. If introduced range monk parakeets switched to emphasizing group membership information in contact calls, then hierarchical mapping patterns in introduced range monk parakeet contact calls should have exhibited stronger convergence at a higher social scale. We used yellow-naped amazons as a baseline for comparison because this species exhibits strong acoustic convergence at a higher social scale with regional vocal dialects that are audibly and visibly distinctive to humans [23,30,31,94]. We found that hierarchical mapping patterns were similar between native and introduced range monk parakeets, supporting our conclusion that the salient type of identity information in monk parakeet contact calls did not change after social disruption that occurred over ecological timescales. In this comparative analysis, we used a customizing bootstrapping approach that yielded similar results for native range and introduced range monk parakeets as our analyses with Earth Mover’s Distance and Mantel tests.

Our comparative analysis also highlighted the importance of using quantitative tools to complement human perception of audible and visible variation in avian vocalizations. When relying on the human ear and eye, the variation among regional dialects in yellow-naped amazon contact calls is far more perceptible than individually distinctive monk parakeet contact calls. For example, the regional dialects that we recapitulated in the amazon contact calls are distinctive to the human ear [23], including North dialect contact calls that sound like “wah-wah”, and variants of the South dialect that sound like “weeup”. In contrast, patterns of individual variation in monk parakeet contact calls are difficult to distinguish by the human ear, and contact calls of different individuals all sound like “chees”. However, when we used quantitative methods to compare hierarchical mapping patterns between species, we found that individual scale convergence in native and introduced range monk parakeet contact calls was stronger than regional dialect convergence for yellow-naped amazons (Fig 6A: panels i, ii, and vii).

Amazon vocal dialects may be more perceptible to humans than monk parakeet individual vocal signatures because of humans’ limited abilities to perceive fine-scale temporal variation at higher frequencies [95,96]. Parrots’ auditory perception abilities appear tuned for higher frequencies, such as orange-fronted conures (*Eupsittula canicularis*), which display the greatest auditory sensitivity in a frequency band that overlaps with the greatest spectral energies in contact calls [97]. In addition, yellow-naped amazon contact calls exhibit slower frequency modulation patterns that are more perceptible to humans, and can also be arranged into fewer categories (e.g. a few regional dialects), a task that should pose reduced cognitive challenges compared to categorizing monk parakeet contact calls by many different individuals [1,98]. Overall, our results from this comparative analysis point to the importance of using computational approaches to identify information in animal signals that is difficult for humans to perceive but may be critical in animal communication systems.

### 4.3 Future research considerations with hierarchical mapping patterns

We combined computational tools with a conceptual framework of how hierarchical mapping patterns are connected to identity signaling in animal vocal signals. This combined approach allowed us to quantify hierarchical mapping patterns and then infer the most salient identity information encoded in vocal signals. Similar computational approaches could be applied to quantify hierarchical mapping patterns with existing datasets of animal signals to learn more about the social environments in which individuals communicate across a broader range of taxa, without depending on the time-intensive collection of social data from marked individuals. When communication signals are learned, hierarchical mapping patterns should capture overall patterns of acoustic variation that represent both active convergence or divergence within social groups, as well as the side-effects of learning from others in a given social group (e.g. vocalizations can be similar when individuals learned from templates that happened to be similar). Here, we used the social scale with the strongest acoustic convergence to infer which type of identity information animals are actively encoding in learned vocalizations (e.g. the type of identity information that is most important to communicate). In our conceptual framework, we considered stronger acoustic convergence as active convergence, and weaker patterns of acoustic convergence as stochastic outcomes associated with learning. For instance, monk parakeet contact calls recorded at the same site did display a degree of convergence (Table B in S1 Appendix), albeit minimal, which should be expected when animals are learning to sound different from others and are learning from the same social group or set of templates.

Whether and how animals perceive and use stronger or weaker patterns of acoustic convergence in learned vocalizations can be assessed experimentally using playbacks of contact call variants. Indeed, the hierarchical mapping patterns identified for a particular population or species can be used as an important foundation for designing biologically relevant playback experiments, which can be more time-consuming than recording communication signals, and are fundamental to understanding how receivers use the information that signalers communicate. Playback experiments are important because mismatches can occur between the social information encoded in signals and the information that receivers use for social recognition, especially when it is cognitively costly to track certain types of information [3,99]. Addressing how different types of identity information are used by receivers will be important, since distantly related avian taxa, including vulturine guineafowl (*Acryllium vulturinum*) and superb fairy-wrens (*Malurus cyaneus*), exhibit multilevel social structures in the wild, suggesting that hierarchical social structures may be more taxonomically widespread than traditionally thought [100,101].

While quantifying hierarchical mapping patterns can yield exciting insights into the identity information that may be important to communicate, researchers should be careful when using these patterns to inform new research directions about identity signaling and social systems. Recording unmarked individuals in natural populations provides only a snapshot of dynamic social interactions, as well as the social information conveyed in signals that is important in a given social environment. For instance, sampling a few vocalizations per individual over a short time frame makes it difficult to assess how identity information encoding may change during dynamic social interactions, such as the rapid vocal matching exhibited by wild orange-fronted conures and rose-breasted cockatoos (*Eolophus roseicapillus*) [36,38,39]. In addition, while the literature has focused on social recognition in more complex social environments with larger social groups and repeated interactions among many individuals [6,12,28,29,99], future work could also address how learned identity signals should change in social environments characterized by fewer individuals and differentiated relationships overall.

## 5. Conclusions

We used native and introduced range monk parakeet contact calls to test whether the type of identity information encoded in learned vocalizations changed in populations that were established after social disruption that occurred over the last 50 years. We used computational tools, including supervised machine learning, to quantify and compare hierarchical mapping patterns in contact calls between the native and introduced ranges. We inferred that identity information encoding was robust to social disruption over short ecological timescales. By comparing hierarchical mapping patterns between monk parakeet and yellow-naped amazon contact calls, we found that identity information encoding in learned parrot vocalizations changed over longer evolutionary timescales. Our results suggest that signaling systems facilitated by socially learned vocalizations can be robust to changes in social conditions over short timescales, despite the flexibility generally attributed to socially learned behaviors. Taken together, our findings point to exciting new research directions on the flexibility or robustness of socially learned communication signals over short evolutionary timescales.

## Supporting information

S1 Appendix Supplementary information about our sampling and analytical pipelines. This document provides more details about the datasets that we used as well as each of our customized analytical pipelines with monk parakeet and yellow-naped amazon contact calls. This appendix also contains Tables A through E.
(PDF)

S1 Fig Similar patterns of acoustic convergence at the individual scale for native and introduced range monk parakeets using spectrographic cross-correlation (SPCC). All 4 panels show SPCC acoustic space generated by multidimensional scaling (MDS) for contact calls of repeatedly sampled monk parakeets in each of the native and introduced ranges. Top left panel: 4 native range individuals that were used to train supervised random forests models. Bottom left panel: 4 introduced range individuals that we used to train supervised random forests models. Top right panel: 4 native range individuals were used to validate supervised random forests models. Bottom right panel: 5 introduced range individuals that were used to validate supervised random forests models. Blue palettes correspond to the native range and gold-brown palettes to the introduced range. In each panel, points represent different calls per repeatedly sampled individual. Individual identities are displayed through shapes and hues per range, and convex hull polygons demonstrate the area encompassed per individual in acoustic space. The acoustic space across all 4 panels can be interpreted on the same axes. Here, individuals were overdispersed in acoustic space, pointing to strong individual signatures in each range. These results were similar to our findings with random forests similarity (Fig 2).
(TIFF)

S2 Fig Low acoustic convergence at the site scale in each range, as well as across the 3 site scale datasets used to address potential repeated sampling of individuals. Plots of random forests acoustic space are shown by similarity method (columns), as well as the three datasets used to address repeated individual sampling in each of the native and introduced ranges (rows). Acoustic space for the clustering and visual classification datasets were generated by filtering multidimensional scaling (MDS) coordinates for the full dataset of calls. The 4 sites shown here and the aesthetics used per range are the same as in Fig 3 in the main text.
(TIFF)

S3 Fig Earth Mover’s Distance individual scale results were consistent across total bin numbers in each of the native and introduced ranges. These results were calculated using spectrographic cross-correlation similarity. The means and 95% confidence intervals (CIs) were obtained by summarizing across 100 resampling iterations for each of the 6 total bin numbers. The calculation used to report results in the main text (16 bins) is shown as a red “X”. The 95% CIs are small and are not visible around the mean.
(TIFF)

S4 Fig Earth Mover’s Distance site scale results were consistent across total bin numbers in each of the native and introduced ranges. These results were generated using spectrographic cross-correlation and random forests similarity, as well as the three site scale datasets used to address repeated sampling of unmarked individuals. The means and 95% confidence intervals (CIs) were obtained by summarizing across 100 resampling iterations for each bin number. The calculation used to report results in the main text (16 bins) is shown as a red “X”. The 95% CIs are small and are not visible around the mean.
(TIFF)

S5 Fig Earth Mover’s Distance site scale results were consistent across total bin numbers over 3 sampling years for Austin, TX (in the U.S. introduced range). These results were generated using spectrographic cross-correlation and random forests similarity, as well as the three site scale datasets used to address repeated sampling of unmarked individuals. The means and 95% confidence intervals (CIs) were obtained by summarizing across 100 resampling iterations for each bin number. The calculation used to report results in the main text (with 16 bins) is shown as a red “X”. These 95% CIs are also small and are not visible around the mean.
(TIFF)

S6 Fig Earth Mover’s Distance site scale results were consistent across total bin numbers over 2 sampling years for New Orleans, LA (in the U.S. introduced range). These results were generated using spectrographic cross-correlation and random forests similarity, as well as the three site scale datasets used to address repeated sampling of unmarked individuals. The means and 95% confidence intervals (CIs) were obtained by summarizing across 100 resampling iterations for each bin number. As above, the calculation used to report results in the main text (with 16 bins) is shown as a red “X”, and the 95% CIs are not visible around the mean.
(TIFF)

S7 Fig The frequency of introduced range monk parakeet sightings relative to other species reported in complete eBird checklists remained low over our sampling years in Austin and New Orleans (2004 to early 2020). Each bar represents the mean percentage of monk parakeets observed relative to other species, averaged across weeks per year. The error bars denote the standard error. Gold rectangles highlight the sampling years in which monk parakeets were recorded in each city.
(JPEG)

S8 Fig Density curves of spectrographic cross-correlation (SPCC) values for monk parakeets and yellow-naped amazons, as well as an acoustic space plot for yellow-naped amazons. Panels A, B, and C show density curves of SPCC values for native range monk parakeets, introduced range monk parakeets, and yellow-naped amazons, respectively. Each density curve was generated from the full symmetric matrix of similarity values for the given species and range (including the diagonal). Panel D shows acoustic space for yellow-naped amazon contact calls, and points are colored by three regional dialects reported in Costa Rica by [23] (Nor = North, Nic = Nicaragua, Sou = South). We used these graphics to doublecheck the similarity values that we used for our comparative analysis.
(JPEG)
