# Supplementary material for: Individual identity information persists in learned calls of introduced parrot populations
Source: PLoS Comput Biol. 2023 Jul 27;19(7):e1011231. doi: 10.1371/journal.pcbi.1011231 (PMC10374045; doi:10.1371/journal.pcbi.1011231)
Supplement: S1 Appendix — This document provides more details about the datasets that we used as well as each of our customized analytical pipelines with monk parakeet and yellow-naped amazon contact calls. This appendix also contains Tables A through E. (PDF) [file pcbi.1011231.s001.pdf]

The annotated code and knitted RMarkdown files supporting the accompanying article are available on GitHub: https://github.com/gsvidaurre/identity-information-post-introduction.

*1. Summary statistics across social scales and ranges*

We described contact call recording and pre-processing steps for both native and introduced range calls in previous work [1,2]. For this research, we used a dataset of contact calls across social scales and ranges that contained 1582 calls. The individual scale dataset contained calls from individuals repeatedly sampled over short timescales (typically a few minutes), while the site scale dataset contained a single call for each “unique” individual. Unless otherwise specified, throughout these materials we used “site” to refer to the nesting sites at which recording sessions were conducted. We sampled some introduced range sites in more than one year. While harmonizing site codes for introduced range sites recorded over time in Austin (based on spatial proximity), we merged 2 pairs of native range sites into a single site each (PFER-01 and PFER-03 were merged into PFER, while RIAC-01 and RIAC-02 were merged into RIAC). These recording sites had been kept separate in previous work to assess fine-scale patterns of geographic variation [1]. This merging yielded 2 fewer sites in the call dataset for native range site scale compared to previous work [1]. See the knitted RMarkdown reports and scripts provided in the GitHub repository associated with this paper, as well as Tables A3 – A5 in [2] for more information about site names, localities, geographic coordinates, and the number of calls sampled per individual and site.

The variation in geographic distances among the sites that we used to compare hierarchical mapping patterns between ranges are reported in the main text. For our temporal analyses in the introduced range, we compared calls recorded in 2004 among 3 sites recorded in Texas (5.59 – 15.79 km apart), as well as 3 sites in Louisiana (4.09 – 10.43 km

apart). We also compared calls from 5 sites recorded in Texas in 2011 (distances of 0.75 – 7.03 km), and then compared calls from 2 sites in Louisiana in 2011 that were 1.19 km apart. For our third temporal sampling comparison in 2019, we used 6 sites in Texas that were 1.56 – 7.95 km apart.

*2. Overview of repeated sampling of unmarked individuals*

We obtained the majority of contact calls in this study from unmarked individuals. At the site scale, we obtained a single contact call per unmarked bird. Therefore, some calls in the site scale dataset may have represented repeated sampling of one or more unmarked individuals, which could influence resulting patterns of acoustic variation at the site scale in each range. We addressed this issue by using three data pre-processing approaches that accounted for repeated sampling of individuals and yielded different versions of the site scale dataset. First, we used the full site scale dataset per range, which represented no filtering for potential repeated sampling of individuals. Next, we identified site scale calls that were most likely to represent repeated sampling of individuals per site-year in each range, and used two approaches (clustering and visual classification) to identify calls from repeated sampling of individuals. Throughout the following sections, the term “site-year” was used to account for the fact that some introduced range sites were recorded in different years, and calls for such sites were not combined across years during the analyses discussed below.

*3. Accounting for repeated sampling of unmarked birds at the site scale*

Although we considered each call in our site scale dataset representative of a “unique” individual, some calls may have instead represented repeated sampling of the same unmarked individuals within sites. After preliminary visual inspection, we suspected that

repeated individual sampling was higher in the introduced range. We reasoned that keeping or removing calls that represented repeated individual sampling could change the patterns of call convergence that we identified at the site scale. To address this concern, we used three approaches to yield different versions of the site scale dataset that allowed us to determine how repeated sampling of individuals may have influenced the patterns of acoustic convergence that we reported at the site scale.

In our first approach, we used all calls recorded per site (e.g. the full site scale dataset), which addressed the possibility that removing similar calls could lead us to underestimate acoustic convergence at the site scale. Since including calls of repeatedly sampling individuals could lead us to instead overestimate acoustic convergence at the site scale, we used two distinct approaches to identify calls that were likely to represent repeatedly sampled individuals (S1 Appendix section 4). We used Gaussian mixture modeling on the SPCC similarity matrix to cluster calls, and we considered each cluster a repeatedly sampled individual. We also presented the clustered calls to six observers in a visual classification interface we designed in RShiny, a platform for dynamic web applications [3]. Observers visually inspected and modified the assignment of calls to clusters (e.g. potential repeated individuals) in the Shiny app. We used results from each of our clustering and visual classification approaches, respectively, to filter out calls attributed to repeatedly sampled individuals, which yielded 2 smaller versions of the site scale dataset. To filter calls, we randomly sampled one call to keep for each repeated individual that was identified per site by either clustering or visual classification, and we also kept all calls per site that were not attributed to repeated individuals.

Our 3 approaches to address repeated sampling of unmarked individuals within sites yielded 3 versions of the site scale dataset: (a) the full dataset, and datasets filtered to

remove the calls that we attributed to repeatedly sampled individuals by either our (b) clustering or our (c) visual classification approaches. We used these 3 site scale datasets in subsequent analyses of hierarchical mapping patterns to assess the robustness of our results to the inclusion or the removal of calls of unmarked individuals that may have been repeatedly sampled as “unique” birds. We also assessed the degree of repeated individual sampling that occurred in our call datasets in each range by assessing how many potential repeated individuals were identified and how many calls were attributed to repeatedly sampled individuals by each of our clustering and visual classification approaches.

*4. Preparing to identify calls of repeatedly sampled individuals*

We used the individual scale dataset (e.g. known repeatedly sampled individuals) to identify calls that were most likely to represent repeated individual sampling at the site scale. SPCC similarity values for all repeatedly sampled individuals per range were split into two separate distributions: values within individuals and values among individuals. Here we performed SPCC with warbleR version 1.1.15. These distributions of SPCC values represented pairwise comparisons of an individual’s calls to its own calls, versus calls of other individuals. We obtained these values through comparisons among individuals recorded at the same site-year. Some lone individuals were represented by only “within” values if no other birds were recorded for the same site-year.

We used the degree of overlap between these distributions to identify a threshold that split SPCC values into each distribution (e.g. within individual versus among individual comparisons) better than expected by chance. This SPCC value was representative of a split between pairwise comparisons within and among known repeatedly sampled individuals, and could therefore be used to identify pairwise comparisons more likely to represent repeated

sampling of individuals in the site scale dataset. Each SPCC distribution was binned by iterating over different numbers of total bins, as the number of total bins could influence the degree of overlap between distributions. The first bin that yielded less than 50% overlap (less than chance) between distributions was identified. We used the minimum SPCC value of this bin as a similarity threshold (0.4384) to identify pairwise comparisons greater than or equal to this threshold in the site scale dataset. In other words, we used pairwise call comparisons that met this condition in the site scale dataset to identify calls that were likely to represent repeated sampling of unmarked individuals.

*5. Identifying calls of repeatedly sampled individuals*

In our first approach to identify repeated sampling of individuals at the site scale, we used Gaussian mixture modeling as a clustering method on SPCC values per site-year and range. We performed clustering with the package mclust version 5.4.1 [4]. Each resulting cluster per site-year was considered a potential repeated individual. Here, we did not set the number of mixture components (e.g. the number of clusters) but rather allowed models to identify the "best" number of clusters, as we did not have *a priori* expectations for the number of potential repeated individuals present per site-year.

In our second approach, we designed a dynamic visual classification interface with RShiny, a platform for dynamic web applications [3]. In this custom-designed visual classification app, we used the number of clusters identified by Gaussian mixture models per site-year as classes to present spectrograms to users. Spectrograms of calls were presented on a separate page per site-year, and were grouped by cluster on each page. The purpose of this visual classification approach was to provide finer-scale control over how repeated individuals were identified compared to Gaussian mixture models. From preliminary visual

inspection of the calls clustered together by Gaussian mixture models, we found that these models often lumped together visibly distinctive calls in the same cluster, and also assigned visibly similar calls across different clusters. For both of these approaches to identify calls of repeatedly sampled individuals (clustering alone, or clustering followed by visual classification), we used an earlier version of the site scale dataset with 1596 calls that included 14 calls from known repeatedly sampled individuals (e.g. 1 call each for 14 native and introduced range individual recorded at sites included in the site scale dataset, such that 14 calls were duplicated across the individual and site scale datasets). We dropped these 14 calls for our main analyses so we could perform multidimensional scaling on all calls across social scales for call similarity measurements.

In our visual classification approach, six observers (including the 5 co-authors) were prompted to assess visible patterns of structural similarities and differences among calls. Observers could decide whether to 1) leave class assignments unchanged, 2) move spectrogram images among classes if they disagreed with how calls had been grouped, or 3) create new classes that represented additional repeated individuals. Observers could also indicate which calls they did not think represented repeated sampling of individuals. Call metadata, including geographic information, was hidden from observers and each observer had to pass a training module (1 native and 2 introduced site-years with known repeatedly sampled individuals) in which they had to identify known repeatedly sampled individuals with 75% classification accuracy, slightly higher than the individual scale classification accuracy reported in previous work [1]. This multi-observer approach split the visual classification task across co-authors. Native and introduced site-years were randomly split as evenly as possible across observers (12 – 16 site-years assessed per observer).

We settled on this multi-observer approach to divide the otherwise time-intensive workload of visually classifying calls for 63 site-years. In addition, native range and 2004 introduced range contact calls had each already been pre-processed by a different observer to address potential repeated sampling of unmarked individuals [1,5]. We felt that a multi-observer approach was important to minimize bias that could arise from a single (and different) observer performing visual assessment of potential repeated individuals per range. In our multi-observer visual classification approach, four sites were evaluated by each observer to facilitate analysis of multi-observer reliability. These four sites were chosen to represent ranges, numbers of calls per site-year, and temporal differences in the introduced range as evenly as possible. However, since native range sites typically had fewer calls sampled than introduced range sites, and only introduced range sites were sampled over time, we used 1 native and 1 introduced range site with 25 calls each, and 1 introduced range site sampled in 2 years with > 50 calls for multi-observer reliability analyses.

*6. Multi-observer reliability of visual classification*

Observers could redo the training module until they achieved high overall training accuracy. While checking the training data, we realized that the training data for one observer was only partially saved. However, the mean ± standard error (SE) training accuracy for the final training run across all 6 observers (78.45% ± 3.73%) was similar to training accuracy for the remaining 5 observers: 78.33% ± 3.97%. We then parsed each observer's prediction data (e.g. all site-years assessed following training) for validation analyses. First, we carried out multi-observer reliability analyses in which we evaluated how many potential repeated individuals were identified at the 4 sites classified by all observers. Second, we calculated the mean ± SE of the number of potential repeated individuals identified per site-year per range,

and the number of calls attributed to these individuals by both methods used to assess repeated individual sampling (clustering via Gaussian mixture models, and visual classification). These statistics allowed us to assess the degree of repeated individual sampling identified per range, and we calculated these statistics after removing the 4 sites used for multi-observer reliability. We identified high variability in how many potential repeated individuals were identified per site-year across observers. Fleiss' kappa, a measurement of multi-observer reliability calculated with the package irr version 0.84.1 [6], was both negative and very small (-0.035). This low kappa value reflected low agreement among observers, and overall, the difficulty of identifying potential repeated individuals per site without *a priori* information. See the knitted RMarkdown reports in the GitHub repository associated with this paper.

*7. Generating site scale datasets for later analyses*

We used our analysis of repeated individual sampling at the site scale to generate 3 versions of the site scale dataset. We used the full set of calls as the first site scale dataset (this contained all calls per site for 1177 calls total). To obtain our second site scale dataset, we filtered the full set of site scale calls by potential repeated individuals identified in our clustering approach with Gaussian mixture models. To do so, we randomly selected one call per potential individual (i.e. cluster) identified per site. We retained all calls for potential individuals for which only a single call had been assigned, as well as calls below the SPCC threshold that were not used in the clustering analyses (515 total calls). To yield the third dataset of the site scale calls, we randomly selected one call per potential individual identified per site in the visual classification data obtained across observers. We also retained calls that had been attributed to "unique" individuals by observers, as well as calls for potential

repeated individuals that had been assigned only a single call, and calls below the SPCC threshold (e.g. calls not used in the visual classification app) per site (618 calls total). For the 4 sites assessed by all observers (S1 Appendix sections 5 and 6), we randomly selected data from one observer per site to retain in the final site scale visual classification dataset. Finally, we identified 4 calls for one repeatedly sampled individual in the native range that had been inadvertently retained for one site at the site scale in previous work [1]. Neither the retention nor removal of these 4 calls affected the Mantel test results reported in [1], but we nevertheless dropped these calls from the site scale dataset (see the associated code on GitHub).

*8. Obtaining acoustic and image measurements for supervised machine learning*

We used supervised random forests models and spectrographic cross-correlation (SPCC) as complementary similarity measurements. Here we describe our random forests modelling approach in more detail. We obtained a large number of acoustic and image measurements to assemble a set of predictors while building supervised random forests models. These acoustic measurements included call similarity measurements as well as several other types of acoustic measurements.

To obtain different call similarity measurements, we measured SPCC on spectrograms and Mel-frequency cepstral coefficients (MFCCs, which reflect how energy is allocated across different frequency bands), dynamic time warping (DTW) on spectral entropy and dominant frequency time series (each estimated at 100 timepoints per call), and multivariate DTW on spectral entropy and dominant frequencies with the warbleR and dtw packages [7,8]. We performed SPCC on spectrograms and MFCCs using a Hanning window, window length of 378, window overlap of 90, a bandpass filter of 0.5 to 9kHz, and 40 warped spectral bands for

MFCC cross-correlation. We performed cross-correlations with warbleR version 1.1.27 in R version 4.1.2 [7,9]. Unless otherwise specified, we used the same settings and package versions for the majority of measurements used as predictors for supervised machine learning.

We also obtained a standard set of 27 spectral acoustic measurements (including duration and frequency measurements, as well as kurtosis and skew [7]), 88 descriptive MFCC statistics (performed with 12 total cepstral coefficients and 40 warped spectral bands), and over 2000 spectrogram image measurements obtained from the software WNDCHRM version 1.6 [1,10]. The MFCC descriptive statistics included the mean, median, minimum, maximum, kurtosis, skewness, and variance of each cepstral coefficient, as well as the mean and variance of the first and second derivatives. The spectrogram image measurements we used are described in more detail in the accompanying code and in [10]. To generate spectrograms for image measurements, we used the warbleR package [7] and the Fourier transformation parameters described above.

*9. Deriving features for supervised machine learning*

We derived acoustic features from the acoustic similarity matrices and the other datasets of acoustic measurements. We used these features as predictors for random forests models, which allowed us to reduce collinearity among the original measurements, and also allowed us to convert similarity matrices to a lower-dimensional, tabular format compatible with random forests models.

To derive acoustic features from acoustic similarity matrices, we applied non-metric multidimensional scaling (MDS) to each similarity matrix to yield low-dimensional solutions with 30 dimensions total. We implemented MDS with the package MASS version 7.3-51.6 [11]

over 1000 maximum iterations. We derived acoustic features from 5 similarity matrices: 1) SPCC with spectrograms, 2) SPCC with MFCC, 3) dynamic time warping (DTW) on spectral entropy timeseries, 4) DTW with dominant frequency time series, and 5) multivariate DTW with spectral entropy and dominant frequencies. Each matrix represented the full dataset of calls across the individual and site scales in each of the native and introduced ranges.

Next, we obtained features from acoustic measurements by applying principal components analysis (PCA) to each set of measurements. We pre-processed measurements prior to running PCA by using the caret package version 6.0-80 [12] to perform Yeo-Johnson transformations, centering and scaling, and to remove variables with near zero variance. PCA was performed with the base package stats in R version 3.4.4 [9]. We derived acoustic features using PCA from the following 3 acoustic measurement datasets: 1) standard spectral acoustic measurements, 2) MFCC descriptive statistics, and 3) spectrogram image measurements. MDS and PCA features were generated for the full dataset of calls across the individual and site scales for both native and introduced range monk parakeet contact calls.

We checked collinearity among all of these features with a Pearson's correlation analysis. We also checked for high correlations between features and signal to noise ratio, but found no relationships with Pearson's $r > 0.75$. We dropped features with Pearson's $| r |$ greater than 0.75 in the collinearity analysis, yielding 1844 predictors for machine learning. These predictors included 30 features each for SPCC on spectrograms, SPCC on MFCC, DTW on dominant frequency contours, DTW on spectral entropy measurements, and multivariate DTW (150 features total obtained by MDS), as well as 25 features for standard spectral acoustic measurements, 87 features for MFCC, and 1582 image features (1694 total features obtained by PCA).

*10. Splitting calls into datasets for supervised machine learning*

As in previous work, we used individual scale calls for random forests model training and validation in a classification approach, in which class labels were individual identities [1]. We used calls from 4 native range and 4 introduced range repeatedly sampled individuals for training (156 calls total, 68.12% of the individual scale dataset). The remaining 4 native range and 5 introduced range repeatedly sampled individuals were used for model validation (73 calls total, 31.88% of the individual scale dataset), and we reserved all of the 1353 site scale calls for prediction. We obtained site scale similarity measurements by both SPCC and random forests, but we used just SPCC similarity for individual scale statistical analyses, as we had used the dataset of individual scale calls for random forests training and validation.

*11. Model training, validation, and prediction*

We trained and tuned a random forests model with the full set of 1844 predictors over 5 iterations of repeated 5-fold cross-validation using caret version 6.0-86 [12] and ranger version 0.12.1 [13]. We performed supervised machine learning analyses in R version 3.6.3, and again in R 4.1.2 (results in the main text are reported from this later run) [9]. The mtry parameter, or the number of predictors randomly selected at each split, was tuned to 186 for the first model built with 2000 trees. We did not vary the number of total trees, as training accuracy showed little change in response to the size of the forest in previous work [1]. After evaluating training performance, we visualized variable importance to confirm that features we expected to be important for classifying calls back to individuals (e.g. SPCC on spectrograms, see the accompanying code) were indeed among the top important features. Cross-validated training accuracy (with a 95% CI) for the first model was 91.67% (86.17, 95.49).

Random forests performance can sometimes be improved by retaining only variables of relatively high importance. We used an automated feature selection algorithm from the Boruta package version 7.0.0 [14] to identify the most important features from the first random forests model. We then used these 114 features to build a second random forests model that was trained and tuned in the same way as the first. The second model achieved 97.44% (93.57, 99.30) cross-validated training accuracy. The second model outperformed the first, so we used the second random forests model with 114 predictors, 2000 trees, and mtry of 2 for validation of individual scale call similarity and prediction of site scale similarity.

For model validation, we assessed whether the second random forests model yielded the patterns of over-dispersion that we expected to see among native range individuals in acoustic space. We applied MDS with 2 dimensions over 1000 iterations to the random forests proximity matrix and visualized calls in low-dimensional acoustic space. Our validation graphic indicated high separation among native range individuals in acoustic space. We further assessed whether random forests picked up on individual distinctiveness by using Gaussian mixture models in a clustering approach with the mclust package version 5.4.1 [4], which identified an optimal number of 8 clusters, similar to the 9 total individuals used between ranges. In addition, clustering accuracy for the native range was the same as reported in previous work, with only a single call misclassified for one individual (see RMarkdown files provided). We designed this validation approach to validate the way in which we used random forests for prediction of call similarity with the full site scale dataset, in that we ignored call classifications produced by the model and extracted the resulting proximity matrix as the random forests similarity matrix [1,15–18]. See the code provided on GitHub for more information.

*12. Patterns of acoustic variation over social scales and ranges*

We compared patterns of acoustic variation per social scale and range in two-dimensional space by using non-metric MDS to reduce SPCC and random forests similarity matrices per social scale. MDS was performed for all calls per social scale and per similarity method with the package MASS version 7.3-51.6 using 1000 maximum iterations [11]. We optimized MDS to yield stress values close to 0.05, which we used as a general rule-of-thumb to identify optimal MDS solutions with low stress [19]. To optimize MDS, we performed MDS over 2 to 15 total dimensions for the SPCC similarity matrix for the full set of 1582 calls (all individual and site scale calls), and for the random forests similarity matrix with 1426 calls (which included the individual scale calls that we used to validate our random forests model, and all site scale calls).

We selected a MDS solution per similarity method by identifying the number of total dimensions that yielded low stress values close to 0.05. MDS solutions with stress values close to 0.05 should accurately represent the original patterns of variation present in the high dimensional data in the resulting lower dimensional space [19]. As the MDS solutions with 15 total dimensions yielded low stress values for each similarity method, we used the first 2 dimensions from each solution to visualize hierarchical mapping patterns for each similarity method. Below we show acoustic space built from SPCC similarity for the individual scale calls used for random forests training and validation, respectively (S1 Fig). We also show SPCC and random forests acoustic space for the site scale across the 3 datasets used to address potential repeated sampling of individuals (S2 Fig). Note that MDS was performed per similarity matrix, so patterns of acoustic variation can be interpreted on the same axes within similarity methods (but not between the two similarity methods).

**Table A. Decoding individuals and sites for Fig 2 and Fig 3**

| Social scale | Range | Department or State | Year | Unique numeric ID | Unique ID |
|---|---|---|---|---|---|
| Individual | Native | Colonia | 2017 | Native-1 | NAT-RAW |
| Individual | Native | Colonia | 2017 | Native-2 | NAT-ZW8 |
| Individual | Native | Colonia | 2017 | Native-3 | NAT-UM3 |
| Individual | Native | Colonia | 2017 | Native-4 | NAT-UM5 |
| Individual | Introduced | Texas | 2019 | Introduced-1 | INV-UM9 |
| Individual | Introduced | Texas | 2019 | Introduced-2 | INV-UM10 |
| Individual | Introduced | Texas | 2019 | Introduced-3 | INV-UM16 |
| Individual | Introduced | Texas | 2019 | Introduced-4 | INV-UM17 |
| Site | Native | Colonia | 2017 | Native-1 | INES-08 |
| Site | Native | Colonia | 2017 | Native-2 | PIED |
| Site | Native | Colonia | 2017 | Native-3 | ROSA |
| Site | Native | Colonia | 2017 | Native-4 | LENA |
| Site | Introduced | Texas | 2019 | Introduced-1 | ELEM |
| Site | Introduced | Texas | 2019 | Introduced-2 | INTR |
| Site | Introduced | Texas | 2019 | Introduced-3 | MART |
| Site | Introduced | Texas | 2019 | Introduced-4 | SOCC |

Decoded individual and site identities for Fig 2B and Fig 3B in the main text. See tables A3 – A5 in [2] for full site names and more information about the localities where calls were recorded for these individuals and sites. In Fig 2A, the native range individual used for the individual scale call lexicon was NAT-UM5, while the introduced range individual was INT-UM16. In Fig 3A, the site used for the native range site scale call lexicon was LENA, and the introduced range site was INTR.

*13. Using Earth Mover's Distance to quantify acoustic convergence*

Earth Mover's Distance represents the amount of work that it takes to convert one distribution into another [20]. For this analysis, we separated similarity values into distributions of contact call comparisons of the same or different categories at each social scale. For instance, at the individual scale, we obtained distributions of similarity values representing contact call comparisons within or among individuals. We calculated Earth Mover's Distance with the package emdist 0.3-2 [21] in a histogram-based approach, in which we binned each distribution by a given number of total bins. We used the mean of each bin and the proportion of values assigned to each bin as the location and weights of each bin per distribution in the one-dimensional space. We used functions from the colordistance package 1.1.1. as a guide for developing this code [22]. Since we did not have *a priori* expectations for an optimal number of bins to use, we iterated over six total bin numbers (2, 4, 8, 16, 32, 64) to calculate Earth Mover's Distance. These six bins represented six powers of 2, or a sequence of numbers that represented coarser to finer bin resolution. We calculated Earth Mover's Distance across these total bin numbers using Euclidean distance and 5000 maximum iterations.

We designed a resampling routine to generate Earth Mover's Distance calculations that could be compared across social scales and ranges. In this customized routine with 100 resampling iterations, we randomly sampled 5 exemplars of each category (e.g. individuals or sites) from each of the native and introduced ranges, and also randomly sampled 5 calls for each of these randomly sampled individuals or sites that had 5 or more calls. For the individual scale analyses, this sample size filter excluded one native range bird with 4 calls (NAT-RAW). We used 5 category exemplars and 5 calls per exemplar in order to accommodate the smaller sample sizes at the individual scale, as we had sampled fewer

overall individuals and calls per individual compared to sites at the higher social scale. We used the calls sampled in each iteration to filter values from the given similarity matrix, which yielded 50 similarity values for the distribution of comparisons within categories, and 250 values for the distribution of comparisons among categories. To generate distributions of equivalent sample sizes for each sampling iteration, we randomly sampled 50 values from the distribution of comparisons among categories before calculating Earth Mover's Distance in the histogram-based approach described above. Each random sampling step was performed without replacement. We performed this resampling routine for each social scale in each of the native and introduced ranges. At the individual scale we used SPCC only as a similarity method as described in the main text. For the site scale, we used contact calls from the most recent sampling year per site when sites were sampled over time. For this higher social scale, we also calculated Earth Mover's Distance for each of the native and introduced ranges with each of the 3 datasets that we used to address the effect of inadvertent repeated sampling of individuals, as well as both similarity methods (SPCC and random forests).

Importantly, for this analysis we did not place geographic restrictions while sampling individuals or sites (in contrast to the Mantel tests and comparative analyses performed below). The results we report using Earth Mover's Distance at the individual scale may include convergence among individuals at the individual scale that were sampled in distant geographic locations. In other words, some individuals may have developed individually distinctive calls that were more similar than expected only because they interacted little or never at all, and therefore did not need to sound distinctive from one another. The fact that our Earth Mover's Distance results still displayed greater convergence overall at the individual scale compared to the site scale demonstrates that our approach was robust to such stochastic convergence among individuals. The resampling routine that we designed also

helped us control for variation in the sites in which individuals were repeatedly sampled (some introduced range individuals were sampled at different sites, but all native range individuals were sampled at the same site), and the possibility of non-independent sites.

We used this same resampling approach and histogram-based approach to calculate Earth Mover's Distance as an estimate of the strength of acoustic convergence at the site scale in Austin, TX over 3 sampling years (2004, 2011, 2019) and New Orleans, LA in 2 sampling years (2004 and 2011). However, for these analyses we randomly sampled 2 sites in each resampling iteration (yielding 20 within site comparisons and 25 among site comparisons in each resampling iteration). Therefore, we obtained 20 similarity values from each of the distributions of comparisons within sites as well the comparisons among sites, since we did not always sample at least 5 sites in each city and sampling year. As above, we calculated Earth Mover's Distance using all 3 site scale datasets that we generated to address the possibility of repeated sampling of unmarked individuals at this social scale. For these analyses, the site scale datasets generated by filtering after clustering and visual classification yielded fewer numbers of sites per year that had at least 5 calls or more (compared to the full dataset, see the main Methods section 2.9). For the dataset filtered by clustering, we used 2 sites in 2004, 5 sites in 2011, and 5 sites in 2019 for Austin, and 2 sites in 2004, and 2 sites in 2011 for New Orleans. For the dataset filtered after visual classification, we used 3 sites in 2004, 5 sites in 2011, and 5 sites in 2019 for Austin, and 2 sites in 2004, and 2 sites in 2011 for New Orleans.

In these comparisons of acoustic convergence between ranges and over time in the introduced range, we did not have *a priori* expectations for an "optimal" number of bins to use for the Earth Mover's Distance calculations. Therefore, we assessed whether these calculations were consistent across total bin numbers by calculating means and 95%

confidence intervals (CIs) for each total bin number (e.g. by summarizing across resampling iterations for each number of total bins). Earth Mover's Distance values were consistent across these bin numbers for the individual and site scale in each of the native and introduced ranges (Figures S3 and S4). We show plots of the raw data generated by our resampling routine (e.g. before calculating means and 95% Cis) in the RMarkdown output files that are available on GitHub. We performed the same quality control checks for our analyses in the introduced range over time, and confirmed that Earth Mover's Distance calculations were also consistent across bins in for the two introduced range cities that we sampled over time (Figures S5 and S6). For our main results in each of the range and temporal comparisons, we calculated means and 95% confidence intervals (CIs) for Earth Mover's Distance values across 100 resampling iterations for 16 total bins.

Overall, we found that Earth Mover's Distance values were highest at the individual scale in contact calls for native and introduced range populations (Table B in S1 Appendix). Earth Mover's Distance values at the site scale were an order of magnitude lower than the individual scale values (Table B in S1 Appendix). When we compared Earth Mover's Distance values over time in two introduced range cities, we found evidence of consistently low acoustic convergence at the site scale over time (Table C in S1 Appendix). Earth Mover's Distance values calculated over time remained a similar order of magnitude to the site scale values we calculated for the introduced range populations overall, all of which were an order of magnitude lower than values obtained for the individual scale (Tables B and C in S1 Appendix).

**Table B. Assessing the strength and significance of acoustic convergence over two social scales with Earth Mover's Distance**

| Social scale | Similarity method | Range | Site scale dataset | Mean Earth Mover's Distance and 95% CI[a] |
|---|---|---|---|---|
| Individual | SPCC | Native | - | 0.159 (0.153, 0.164) |
| | | Introduced | - | 0.131 (0.125, 0.138) |
| Site | SPCC | Native | Full | 0.033 (0.031, 0.035) |
| | | | Clustering | 0.030 (0.027, 0.032) |
| | | | Visual classification | 0.030 (0.027, 0.032) |
| | | Introduced | Full | 0.073 (0.068, 0.077) |
| | | | Clustering | 0.051 (0.048, 0.054) |
| | | | Visual classification | 0.049 (0.046, 0.053) |
| | Random forests | Native | Full | 0.020 (0.019, 0.021) |
| | | | Clustering | 0.017 (0.016, 0.019) |
| | | | Visual classification | 0.017 (0.016, 0.019) |
| | | Introduced | Full | 0.036 (0.034, 0.038) |
| | | | Clustering | 0.027 (0.025, 0.028) |
| | | | Visual classification | 0.026 (0.024, 0.027) |

[a]Means and confidence intervals are shown for calculations obtained using 16 total bins for each distribution over 100 resampling iterations.

**Table C. Evaluating temporal change in site scale acoustic convergence in the introduced range**

| Similarity method | City | Year | Dataset | Mean Earth Mover's Distance and 95% CI[a] |
|---|---|---|---|---|
| SPCC | Austin | 2004 | Full | 0.042 (0.039, 0.045) |
| | | | Clustering | 0.038 (0.036, 0.041) |
| | | | Visual classification | 0.029 (0.027, 0.031) |
| | | 2011 | Full | 0.059 (0.053, 0.065) |
| | | | Clustering | 0.045 (0.041, 0.049) |
| | | | Visual classification | 0.051 (0.047, 0.056) |
| | | 2019 | Full | 0.039 (0.036, 0.043) |
| | | | Clustering | 0.032 (0.029, 0.034) |
| | | | Visual classification | 0.034 (0.032, 0.036) |
| | New Orleans | 2004 | Full | 0.065 (0.059, 0.071) |
| | | | Clustering | 0.044 (0.041, 0.048) |
| | | | Visual classification | 0.079 (0.074, 0.084) |
| | | 2011 | Full | 0.042 (0.039, 0.046) |
| | | | Clustering | 0.045 (0.042, 0.048) |
| | | | Visual classification | 0.044 (0.041, 0.046) |
| Random forests | Austin | 2004 | Full | 0.023 (0.021, 0.025) |
| | | | Clustering | 0.023 (0.021, 0.025) |
| | | | Visual classification | 0.017 (0.016, 0.018) |
| | | 2011 | Full | 0.033 (0.030, 0.036) |
| | | | Clustering | 0.025 (0.023, 0.027) |
| | | | Visual classification | 0.028 (0.026, 0.030) |
| | | 2019 | Full | 0.017 (0.015, 0.018) |
| | | | Clustering | 0.015 (0.014, 0.017) |
| | | | Visual classification | 0.013 (0.012, 0.014) |
| | New Orleans | 2004 | Full | 0.031 (0.028, 0.034) |
| | | | Clustering | 0.016 (0.015, 0.018) |
| | | | Visual classification | 0.034 (0.032, 0.036) |
| | | 2011 | Full | 0.017 (0.016, 0.018) |
| | | | Clustering | 0.015 (0.013, 0.016) |
| | | | Visual classification | 0.016 (0.015, 0.017) |

[a]Means and confidence intervals are shown for calculations obtained using 16 total bins for each distribution over 100 resampling iterations.

*14. Population trends in the introduced range*

We addressed the possibility of significant population growth in the introduced range by assessing population trends in two cities where we recorded parakeets over time. We used eBird checklists to assess population trends in Austin, Texas, U.S. and New Orleans, Louisiana, U.S. To perform this analysis, we accessed the eBird Basic Dataset on 16 September 2020 to download checklists for Travis County (TX) and Orleans County (LA), which contained data for the cities of Austin (sampled in 2004, 2011, and 2019), and New Orleans (sampled in 2004 and 2011), respectively [23]. We downloaded data for all species to facilitate calculating the frequency of monk parakeets in checklists over time relative to all other species observed. Data was downloaded from January 2004 to January 2020 for both counties, and we did not include unvetted data. The package auk version 0.4.1 [24] was used to read in eBird data in R version 3.6.3 [9], and we filtered the county scale eBird data to obtain sightings for each city.

We averaged the frequency of monk parakeet sightings in complete checklists relative to all other species over weeks and years to yield an annual percentage of monk parakeet sightings per city. We used this annual percentage of monk parakeet sightings to evaluate population trends over the span of our sampling years [25]. Although we did not directly evaluate changes in local population size over time, we considered the frequency of monk parakeet sightings over time to be an indicator of relative changes in population size over time, as larger populations of monk parakeets should yield more frequent sightings than smaller populations. We calculated the frequencies of monk parakeet sightings relative to all other species reported in complete eBird checklists, to correct for the general trend of increased eBird reporting over time [25]. We found that the annual percentage of monk parakeet sightings, which represented the frequency of monk parakeet sightings relative to

other avian species, remained low in Austin and New Orleans throughout our sampling years (S7 Fig). The mean annual percentage of monk parakeet sightings never rose above 5% relative to all species observed in complete checklists between 2004 and 2019 for either city. See the code and knitted RMarkdown reports provided on GitHub for more information.

*15. Choosing individuals and sites for Mantel tests*

We used Mantel tests to assess hierarchical mapping patterns over social scales for native and introduced range monk parakeet calls. To compare acoustic convergence at the individual scale between ranges, we chose 5 repeatedly sampled individuals recorded in a single year over relatively similar geographic areas per range that captured similar patterns of acoustic variation within versus among individuals [1]. This was important, because we previously documented greater patterns of overlap in acoustic space over increasing geographic distances [1], which could confound the strength of acoustic convergence at the individual scale between ranges.

At the site scale, we quantified hierarchical mapping patterns for native and introduced range monk parakeets, but also for introduced range parakeets over time. For our comparison between native and introduced range parakeets at this higher social scale, we used calls recorded in the most recent sampling year when a site (Austin) or city (New Orleans) was recorded in more than one year. For our temporal comparison, we used calls recorded in the cities of Austin in 2004, 2011, and 2019, as well as calls recorded in the city of New Orleans in 2004 and 2011.

*16. Using Mantel tests to compare hierarchical mapping patterns between the native and introduced ranges*

After evaluating hierarchical mapping patterns in acoustic space, we used Mantel tests to assess acoustic convergence at each social scale for both of the native and introduced ranges. We performed Mantel tests with matrices of call similarity (SPCC for the individual scale; random forests and SPCC for the site scale) and matrices of binary identity per social scale over 9999 permutations. We performed Mantel tests with 9999 permutations over social

scales and ranges using the package vegan version 2.5-6 [26]. These tests allowed us to ask whether calls were more similar within an individual or site compared to among individuals or sites for both the native and introduced ranges.

We conducted Mantel tests with binary matrices of either individual or site identity. We generated these binary matrices by assigning calls compared within the same individual or site the value of 0, and a value of 1 to calls compared among individuals or sites. Call similarity and binary identity similarity matrices were converted to distances by subtracting matrices from 1. We performed a partial Mantel test for the introduced range individual scale using a third matrix of binary site identity, as these individuals were sampled over more than one site. For the site scale, we also ran Mantel tests with the three versions of the site scale dataset used to address repeated sampling of individuals at that social scale. We performed a total of 2 Mantel tests for the individual scale and 12 Mantel tests for the site scale in this comparison of hierarchical mapping patterns between ranges. We adjusted alpha of 0.05 to 0.0036 by a Bonferroni correction to account for 14 tests total. All Mantel test statistics were statistically significant under the Bonferroni-corrected alpha (Table D in S1 Appendix).

**Table D. Assessing acoustic convergence over two social scales with Mantel tests**

| Social scale | Similarity method | Range | Site scale dataset | Number of calls | Number of sites | Mantel *r* | Permuted p-values |
|---|---|---|---|---|---|---|---|
| Individual | SPCC | Native | - | 78 | 1 | 0.48 | 0.0001 |
| | | Introduced | - | 52 | 3 | 0.50 | 0.0001 |
| Site | SPCC | Native | Full | 598 | 37 | 0.06 | 0.0001 |
| | | | Clustering | 410 | 37 | 0.04 | 0.0001 |
| | | | Visual classification | 336 | 37 | 0.04 | 0.0001 |
| | | Introduced | Full | 579 | 18 | 0.20 | 0.0001 |
| | | | Clustering | 208 | 18 | 0.11 | 0.0001 |
| | | | Visual classification | 179 | 18 | 0.11 | 0.0001 |
| | Random forests | Native | Full | 598 | 37 | 0.13 | 0.0001 |
| | | | Clustering | 410 | 37 | 0.10 | 0.0001 |
| | | | Visual classification | 336 | 37 | 0.10 | 0.0001 |
| | | Introduced | Full | 579 | 18 | 0.29 | 0.0001 |
| | | | Clustering | 208 | 18 | 0.24 | 0.0001 |
| | | | Visual classification | 179 | 18 | 0.24 | 0.0001 |

*17. Using Mantel tests to evaluate hierarchical mapping patterns over time in the introduced range*

We also used Mantel tests to assess acoustic convergence at the site scale over time in the introduced range. For these analyses, we used the same Mantel test routine with 9999 permutations described above (S1 Appendix section 16), but we focused on introduced range populations that we had repeatedly recorded in Austin, Texas and New Orleans, Louisiana. For each year that we had sampled calls in each city, we performed a Mantel test to correlate matrices of call similarity against matrices of binary site identity for the sites sampled in a given city and year. We performed Mantel tests per city because we did not always sample the same sites in each year. For Austin, we performed Mantel tests using different sites recorded in each of 3 sampling years: 3 sites in 2004, 5 sites in 2011, and 6 sites in 2019. For New Orleans, we conducted Mantel tests using different sites sampled in 2 years: 3 sites in 2004 and 2 sites in 2011. We conducted Mantel tests with random forests and SPCC similarity measurements, as well as each of the 3 site scale datasets, and adjusted alpha of 0.05 to 0.0017 account for 30 tests total. After adjusting alpha for multiple comparisons, we identified statistically significant Mantel test statistics at the site scale in 2011 in the city of Austin using the full dataset of calls by both similarity methods, and the datasets filtered after clustering and visual classification, but only by random forests similarity (Table E in S1 Appendix). We also found statistically significant convergence within sites in Austin in 2019 and New Orleans in 2004 using the full dataset of calls and both similarity methods (Table E in S1 Appendix).

**Table E. Evaluating site scale acoustic convergence over time in the introduced range with Mantel tests**

| Similarity method | City | Year | Dataset | Number of calls | Number of sites | Mantel $r^a$ | Permuted p-values |
|---|---|---|---|---|---|---|---|
| SPCC | Austin | 2004 | Full | 93 | 3 | 0.10 | 0.0796 |
| | | | Clustering | 37 | 3 | 0.09 | 0.1359 |
| | | | Visual inspection | 20 | 3 | -0.03 | 0.5950 |
| | | 2011 | Full | 65 | 5 | **0.23** | 0.0001 |
| | | | Clustering | 43 | 5 | 0.08 | 0.0067 |
| | | | Visual inspection | 54 | 5 | 0.07 | 0.0055 |
| | | 2019 | Full | 295 | 6 | **0.13** | 0.0001 |
| | | | Clustering | 83 | 6 | 0.01 | 0.2513 |
| | | | Visual inspection | 56 | 6 | -0.03 | 0.8498 |
| | New Orleans | 2004 | Full | 49 | 3 | **0.18** | 0.0005 |
| | | | Clustering | 18 | 3 | 0.03 | 0.3272 |
| | | | Visual inspection | 25 | 3 | -0.02 | 0.6186 |
| | | 2011 | Full | 29 | 2 | 0.27 | 0.0045 |
| | | | Clustering | 17 | 2 | 0.22 | 0.0196 |
| | | | Visual inspection | 17 | 2 | 0.21 | 0.0229 |
| Random forests | Austin | 2004 | Full | 93 | 3 | 0.03 | 0.2680 |
| | | | Clustering | 37 | 3 | 0.03 | 0.2912 |
| | | | Visual inspection | 20 | 3 | 0.04 | 0.3015 |
| | | 2011 | Full | 65 | 5 | **0.37** | 0.0001 |
| | | | Clustering | 43 | 5 | **0.13** | 0.0004 |
| | | | Visual inspection | 54 | 5 | **0.09** | 0.0015 |
| | | 2019 | Full | 295 | 6 | **0.17** | 0.0001 |
| | | | Clustering | 83 | 6 | 0.05 | 0.0099 |
| | | | Visual inspection | 56 | 6 | 0.01 | 0.4115 |
| | New Orleans | 2004 | Full | 49 | 3 | **0.19** | 0.0002 |
| | | | Clustering | 18 | 3 | 0.10 | 0.1161 |
| | | | Visual inspection | 25 | 3 | -0.02 | 0.6051 |
| | | 2011 | Full | 29 | 2 | 0.09 | 0.0826 |
| | | | Clustering | 17 | 2 | 0.08 | 0.1827 |
| | | | Visual inspection | 17 | 2 | 0.07 | 0.1963 |

[a]Mantel *r* values that were statistically significant under the corrected alpha are shown in bold.

*18. Effects of repeated individual sampling and geographic isolation*

While we identified stronger individual signatures than convergence within sites in each range, we also identified generally higher convergence at the site scale for the introduced range compared to the native range (Table B in S1 Appendix). If this difference in the magnitude of site scale convergence between ranges reflected a change in identity information encoding, then we should have consistently identified the strongest acoustic convergence at this social scale in introduced range calls. Instead, we found that the magnitude of site scale acoustic convergence for the introduced range was sensitive to the call dataset that we used (Table B in S1 Appendix). In addition, the strength of convergence that we identified at the site scale for the introduced range was consistently lower than individual scale convergence in each of the native and introduced ranges (Table B in S1 Appendix). Therefore, the higher acoustic convergence that we reported at the site scale for the introduced range may reflect repeated sampling of unmarked individuals within sites, which is further supported by the greater degree of repeated individual sampling in our introduced range site scale dataset (Table 1).

However, the higher convergence that we identified for the introduced range site scale may also reflect the geographic isolation of introduced range populations. Introduced range monk parakeet populations are largely confined to city boundaries in the U.S. [27] and are more geographically disjunct than native range populations that exhibit greater contiguity over large geographic areas (Fig 1; Smith-Vidaurre, pers. obs.). Our field observations in Uruguay (Smith-Vidaurre, pers. obs.) and Argentina [28] indicate that native range parakeets readily interact with individuals from other sites, creating more opportunities for social learning that could blur the boundaries among sites in acoustic space. Parakeets in smaller and less contiguous introduced range populations should be limited to learning from others in their

immediate social groups, yielding more convergence within sites (and more distinctiveness among sites) compared to larger and more contiguous native range populations. Finally, it is possible that simplified individual signatures in introduced range contact calls, or calls that contain less individual identity information [2], also contributed to the higher convergence that we identified for the introduced range site scale. Together, a greater degree of repeated sampling of individuals, lower population contiguity, and simpler individual vocal signatures likely explain the higher acoustic convergence that we identified at the site scale for the introduced range.

*19. Preparing for comparative analyses*

We used previously published calls of yellow-naped amazons (*Amazona auropalliata*), a parrot species that exhibits convergence on calls within sites and regional populations [29] to benchmark the hierarchical mapping patterns we identified for monk parakeets. We matched sampling rates of calls for each species by downsampling monk parakeet calls to 22.05 kHz. Then we performed SPCC for both species with a Hanning window, window length of 378, window overlap of 90, bandpass filter of 0.5 – 9kHz, and Pearson's correlation with warbleR 1.1.27 [7].

We generated summary statistics and validation plots of the full set of SPCC similarity values for each species (S8 Fig) to ground our expectations for a bootstrapping analysis (see below). We confirmed that the mean SPCC value from the full SPCC matrix for native monk parakeet calls was 0.39, while introduced range monk parakeet calls had a mean of 0.30, and the mean value for yellow-naped amazon contact calls was 0.72. These mean calculations included the similarity values from the lower half, upper half, and diagonal of each symmetric SPCC matrix. When we plotted the full SPCC matrix as a density curve for each range and

species, these plots further showed that SPCC values for native and introduced range monk parakeets were centered below 0.5 (panels A and B, S8 Fig), while yellow-naped amazon SPCC values were centered above 0.5 (panel C, S8 Fig). These density curves were also not bimodal (e.g. none of these distributions showed two large peaks at lower and higher SPCC values). In other words, in subsequent analyses, the values that we used for comparisons within or among sites or individuals generally fell within the bounds of the large single peak of SPCC values for each range and species (S8 Fig).

The mean and overall distribution of similarity values for yellow-naped amazons matched the range of SPCC values originally reported in [29]. For additional validation, we also performed MDS with 15 total dimensions on the SPCC matrix for yellow-naped amazon contact calls and generated a plot of low-dimensional acoustic space to evaluate whether we could reproduce results reported in the original 1996 study (panel D, S8 Fig). After confirming that we had reproduced the quantifiable acoustic differences among regional vocal dialects originally reported for yellow-naped amazons in 1996, we moved on to our comparative analysis.

*20. Selecting SPCC values for comparative analyses*

In this analysis, our goal was to quantify and directly compare hierarchical mapping patterns among native range monk parakeets, introduced range monk parakeets, and yellow-naped amazons. We performed SPCC for each species after matching sampling rates of audio files per species (S1 Appendix section 19). For yellow-naped amazons, we used previously published contact calls recorded in Costa Rica in 1994 [29]. We selected SPCC values for a subsample of individuals or groups at each social scale that represented similar sampling depth and geographic breadth for each range and species. We used these subsamples of individuals or groups to compare SPCC values within or among these categories at each social scale.

At the individual scale, we used 5 repeatedly sampled native range monk parakeets recorded at the same site in the department of Colonia, Uruguay in 2017. We chose 5 repeatedly sampled introduced range monk parakeets sampled at 3 sites close to each other in the city of Austin, TX, U.S. in 2019. We decided to use these introduced range individuals because parakeets recorded at more distant sites were more likely to overlap more in acoustic space [2]. For yellow-naped amazons, we chose 12 repeatedly sampled individuals from 3 randomly sampled North dialect sites in Costa Rica.

For the site scale, we selected 6 randomly sampled sites for native range monk parakeets that we had recorded in the department of Colonia, Uruguay in 2017. We chose 6 sites for introduced range monk parakeets that we had recorded in the city of Austin, U.S. in 2019. We chose these particular sites for native and introduced range monk parakeets to obtain similarity values representing call comparisons over a geographic breadth similar to yellow-naped amazons. For yellow-naped amazons, we used 7 sites recorded in the North

dialect geographic region of Costa Rica, which spanned less than 40km in 1994 [29]). Two to four birds were usually repeatedly sampled per site in the original dataset of [29].

We also quantified acoustic convergence at another social scale, the regional dialect scale, for yellow-naped amazons only. We selected calls recorded from the North and South regional dialects in Costa Rica [29] for this analysis. Native range and U.S. introduced range monk parakeets did not exhibit visually distinctive regional vocal dialects in the contact calls that we sampled [1,2,5].

We then selected SPCC values for the subsampled individuals and groups from the full SPCC matrix for native range monk parakeets, introduced range monk parakeets, and yellow-naped amazons. We obtained SPCC values that represented pairwise comparisons among calls within or among these different categories (e.g. within the same individual or group as well as among different individuals or groups). We plotted density curves of these SPCC values within the same category or among different categories across social scales for each species and range (Fig 6A).

In these plots, we used greater separation between the distributions of similarity values representing same versus different category comparisons as an indicator of greater convergence at a particular social scale. Strong convergence occurs when individuals produce calls more similar within than among categories, such that similarity values representing comparisons within the same category should be high, and should also overlap less with similarity values representing comparisons among different categories. For instance, the distributions of similarity values for the individual scale (Fig 6A, panels i and ii) displayed more separation than the site scale for native and introduced range parakeets (Fig 6A, panels iv and v).

*21. Bootstrapping SPCC values for comparative benchmarking analysis*

The main bootstrapping analysis we performed is described in the main text. For native range and introduced range monk parakeets, we also performed this bootstrapping analysis at the site scale across the 3 datasets that we used to account for repeated sampling of unmarked individuals. We found generally similar results at the site scale for native and introduced range monk parakeets, and we reported main results for monk parakeets from the full site scale dataset (Fig 6B). See the code provided on GitHub for bootstrapping analyses and results across monk parakeet site scale datasets.

**References:**

1. Smith-Vidaurre G, Araya-Salas M, Wright TF. Individual signatures outweigh social group identity in contact calls of a communally nesting parrot. Behav Ecol. 2020;31(2):448–58. doi: 10.1093/beheco/arz202

2. Smith-Vidaurre G, Perez-Marrufo V, Wright TF. Individual vocal signatures show reduced complexity following invasion. Anim Behav. 2021;179:15–39. doi: 10.1016/j.anbehav.2021.06.020

3. Chang W, Cheng J, Allaire JJ, Xie Y, McPherson J. shiny: Web application framework for R. 2018.

4. Scrucca L, Fop M, Murphy TB, Raftery AE. mclust 5: Clustering, classification and density estimation using Gaussian finite mixture models. R J. 2016;8(1):289–317.

5. Buhrman-Deever SC, Rappaport AR, Bradbury JW. Geographic variation in contact calls of feral North American populations of the monk parakeet. Condor. 2007;109(2):389–98. doi: 10.1093/condor/109.2.389

6. Gamer M, Lemon J, Singh IFP. irr: Various coefficients of interrater reliability and agreement. R package version 0.84.1. 2019.

7. Araya-Salas M, Smith-Vidaurre G. warbleR: An R package to streamline analysis of animal acoustic signals. Methods Ecol Evol. 2017;8(2):184–91. doi: 10.1111/2041-210X.12624

8. Giorgino T. Computing and visualizing dynamic time warping alignments in R: the dtw package. J Stat Softw. 2009;31(7):1–24. doi: 10.18637/jss.v031.i07

9. R Core Team. R: A language and environment for statistical computing. R Foundation for Statistical Computing, Vienna, Austria; 2022.

10. Shamir L, Orlov N, Eckley DM, Macura T, Johnston J, Goldberg IG. Wndchrm - an open source utility for biological image analysis. Source Code Biol Med. 2008;3:1–13. doi: 10.1186/1751-0473-3-13

11. Venables WN, Ripley BD. Modern Applied Statistics with S. New York: Fourth Edition, Springer; 2002.

12. Kuhn M. Building predictive models in R using the caret package. J Stat Softw. 2008;28(5):1–26. doi: 10.18637/jss.v028.i05

13. Wright MN, Ziegler A. ranger: A fast implementation of random forests for high dimensional data in C++ and R. J Stat Softw. 2017;77(1):1–17. doi: 10.18637/jss.v077.i01

14. Kursa MB, Rudnicki WR. Feature selection with the Boruta package. J Stat Softw. 2010;36(11):1–13. doi: 10.18637/jss.v036.i11

15. Keen S, Ross JC, Griffiths ET, Lanzone M, Farnsworth A. A comparison of similarity-based approaches in the classification of flight calls of four species of North American wood-warblers (Parulidae). Ecol Inform. 2014;21:25–33. doi: 10.1016/j.ecoinf.2014.01.001

16. Humphries GRW, Buxton RT, Jones IL. Machine learning techniques for quantifying geographic variation in Leach's storm-petrel (*Hydrobates leucorhous*). In: Humphries GRW, Magness DR, Huettmann F, editors. Machine Learning for Ecology and Sustainable Natural Resource Management. Cham, Switzerland: Springer Nature; 2018. p. 295–312. doi: 10.1007/978-3-319-96978-7_15

17. Odom KJ, Araya-Salas M, Morano JL, Ligon RA, Leighton GM, Taff CC, et al. Comparative bioacoustics: A roadmap for quantifying and comparing animal sounds across diverse taxa. Biol Rev. 2021;96(4):1135–59. doi: 10.1111/brv.12695

18. Keen SC, Odom KJ, Webster MS, Kohn GM, Wright TF, Araya-Salas M. A machine learning approach for classifying and quantifying acoustic diversity. Methods Ecol Evol. 2021;12(7):1213–25. doi: 10.1111/2041-210x.13599

19. Kruskal JB. Nonmetric multidimensional scaling: A numerical method. Psychometrika. 1964;29(2):115–29. doi: 10.1007/BF02289694

20. Rubner Y, Tomasi C, Guibas LJ. The Earth Mover's Distance as a metric for image retrieval. Int J Comput Vis. 2000;40(2):99.

21. Urbanek S, Rubner Y. emdist: Earth Mover's Distance. 2022.

22. Weller HI, Westneat MW. Quantitative color profiling of digital images with earth mover's distance using the R package colordistance. PeerJ. 2019;7:e6398. doi: 10.7717/peerj.6398

23. Sullivan BL, Wood CL, Iliff MJ, Bonney RE, Fink D, Kelling S. eBird: A citizen-based bird observation network in the biological sciences. Biol Conserv. 2009;142(10):2282–92. doi: 10.1016/j.biocon.2009.05.006

24. Strimas-Mackey M, Miller E, Hochachka W. auk: eBird data extraction and processing with AWK. R package version 0.4.1. 2018.

25. Clark CJ. eBird records show substantial growth of the Allen's Hummingbird (*Selasphorus sasin sedentarius*) population in urban Southern California. Condor. 2017;119(1):122–30. doi: 10.1650/condor-16-153.1

26. Oksanen J, Blanchet FG, Friendly M, Kindt R, Legendre P, McGlinn D, et al. vegan: community ecology package. 2018.

27. Davis AY, Malas N, Minor ES. Substitutable habitats? The biophysical and anthropogenic drivers of an exotic bird’s distribution. Biol Invasions. 2014;16(2):415–27. doi: 10.1007/s10530-013-0530-z

28. Hobson EA, Avery ML, Wright TF. The socioecology of monk parakeets: insights into parrot social complexity. Auk. 2014;131:756–75. doi: 10.1642/AUK-14-14.1

29. Wright TF. Regional dialects in the contact call of a parrot. Proc R Soc London, B. 1996;263:867–72. doi: 10.1098/rspb.1996.0128
