# Supplementary figures and images for: Individual identity information persists in learned calls of introduced parrot populations
Source: PLoS Comput Biol. 2023 Jul 27;19(7):e1011231. doi: 10.1371/journal.pcbi.1011231 (PMC10374045; doi:10.1371/journal.pcbi.1011231)

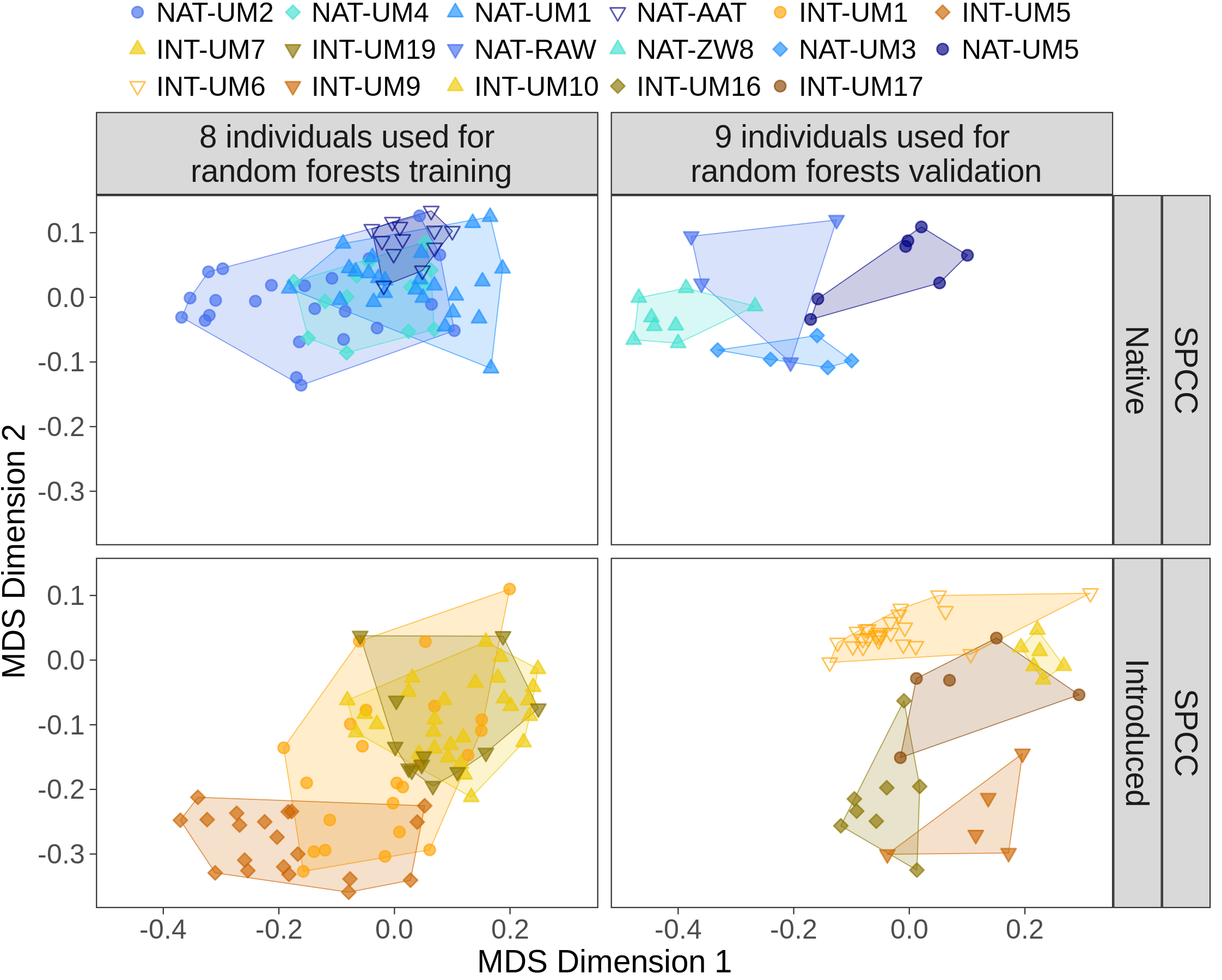

Supplement: S1 Fig — All 4 panels show SPCC acoustic space generated by multidimensional scaling (MDS) for contact calls of repeatedly sampled monk parakeets in each of the native and introduced ranges. Top left panel: 4 native range individuals that were used to train supervised random forests models. Bottom left panel: 4 introduced range individuals that we used to train supervised random forests models. Top right panel: 4 native range individuals were used to validate supervised random forests models. Bottom right panel: 5 introduced range individuals that were used to validate supervised random forests models. Blue palettes correspond to the native range and gold-brown palettes to the introduced range. In each panel, points represent different calls per repeatedly sampled individual. Individual identities are displayed through shapes and hues per range, and convex hull polygons demonstrate the area encompassed per individual in acoustic space. The acoustic space across all 4 panels can be interpreted on the same axes. Here, individuals were overdispersed in acoustic space, pointing to strong individual signatures in each range. These results were similar to our findings with random forests similarity (Fig 2). (TIFF) [file pcbi.1011231.s002.tiff]

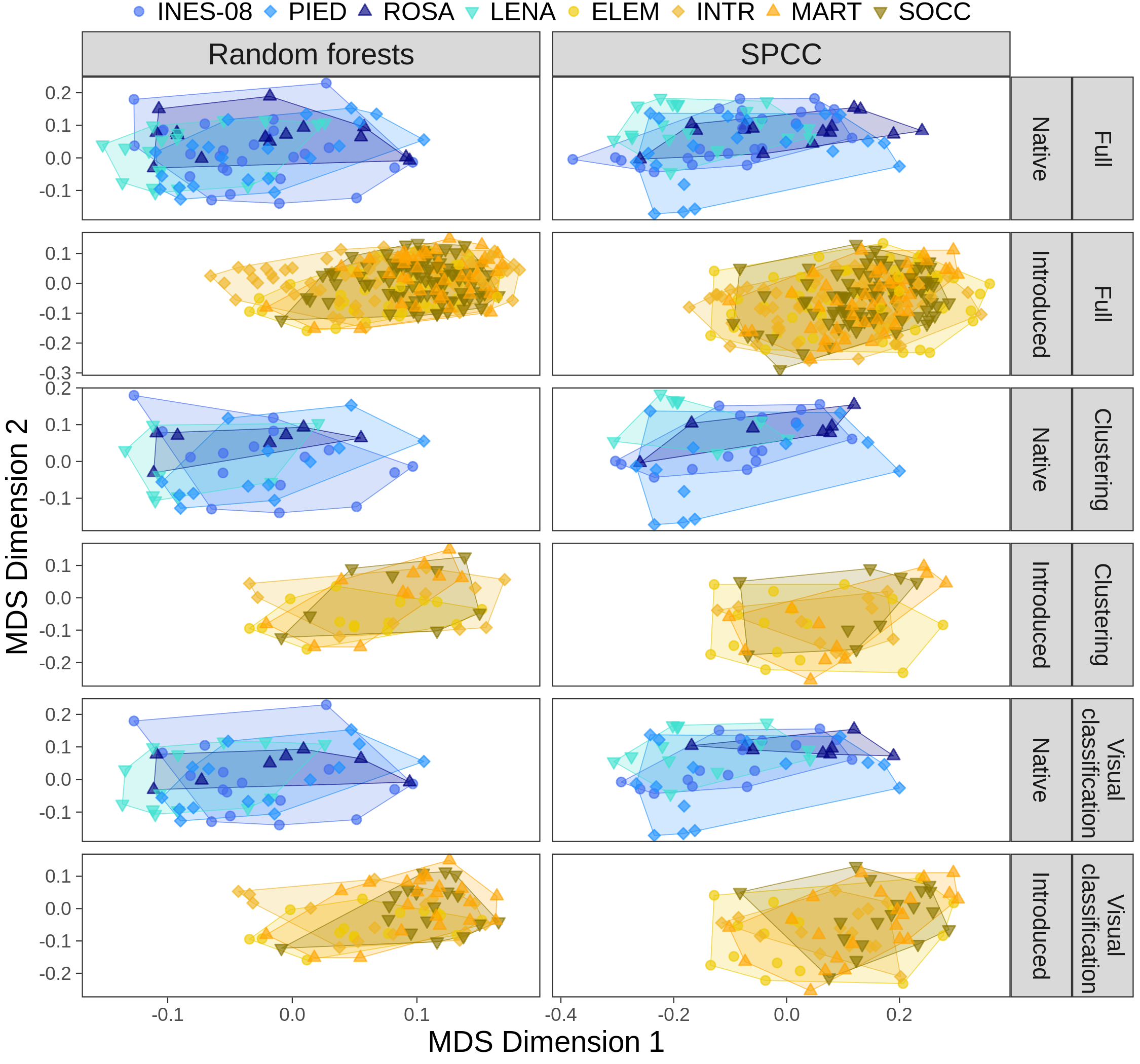

Supplement: S2 Fig — Plots of random forests acoustic space are shown by similarity method (columns), as well as the three datasets used to address repeated individual sampling in each of the native and introduced ranges (rows). Acoustic space for the clustering and visual classification datasets were generated by filtering multidimensional scaling (MDS) coordinates for the full dataset of calls. The 4 sites shown here and the aesthetics used per range are the same as in Fig 3 in the main text. (TIFF) [file pcbi.1011231.s003.tiff]

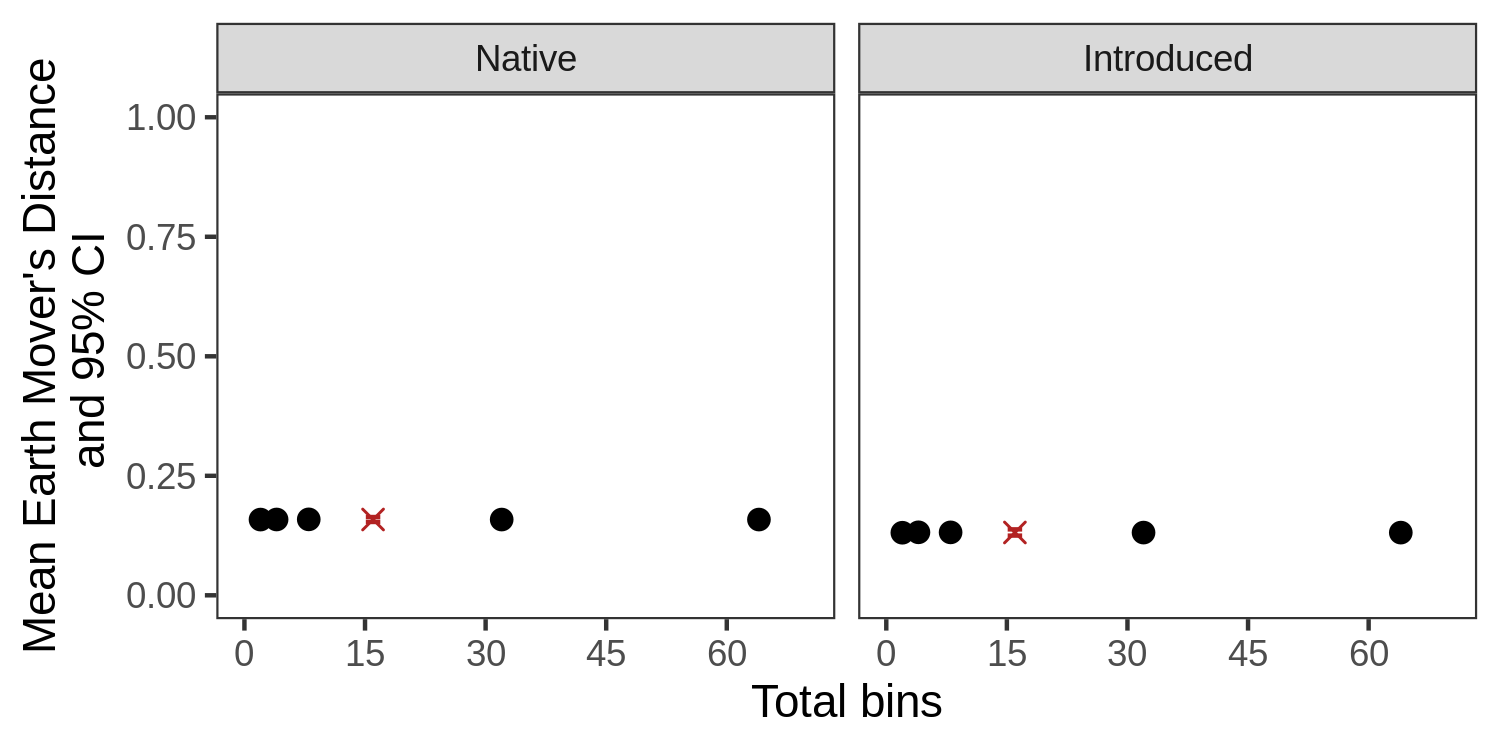

Supplement: S3 Fig — These results were calculated using spectrographic cross-correlation similarity. The means and 95% confidence intervals (CIs) were obtained by summarizing across 100 resampling iterations for each of the 6 total bin numbers. The calculation used to report results in the main text (16 bins) is shown as a red “X”. The 95% CIs are small and are not visible around the mean. (TIFF) [file pcbi.1011231.s004.tiff]

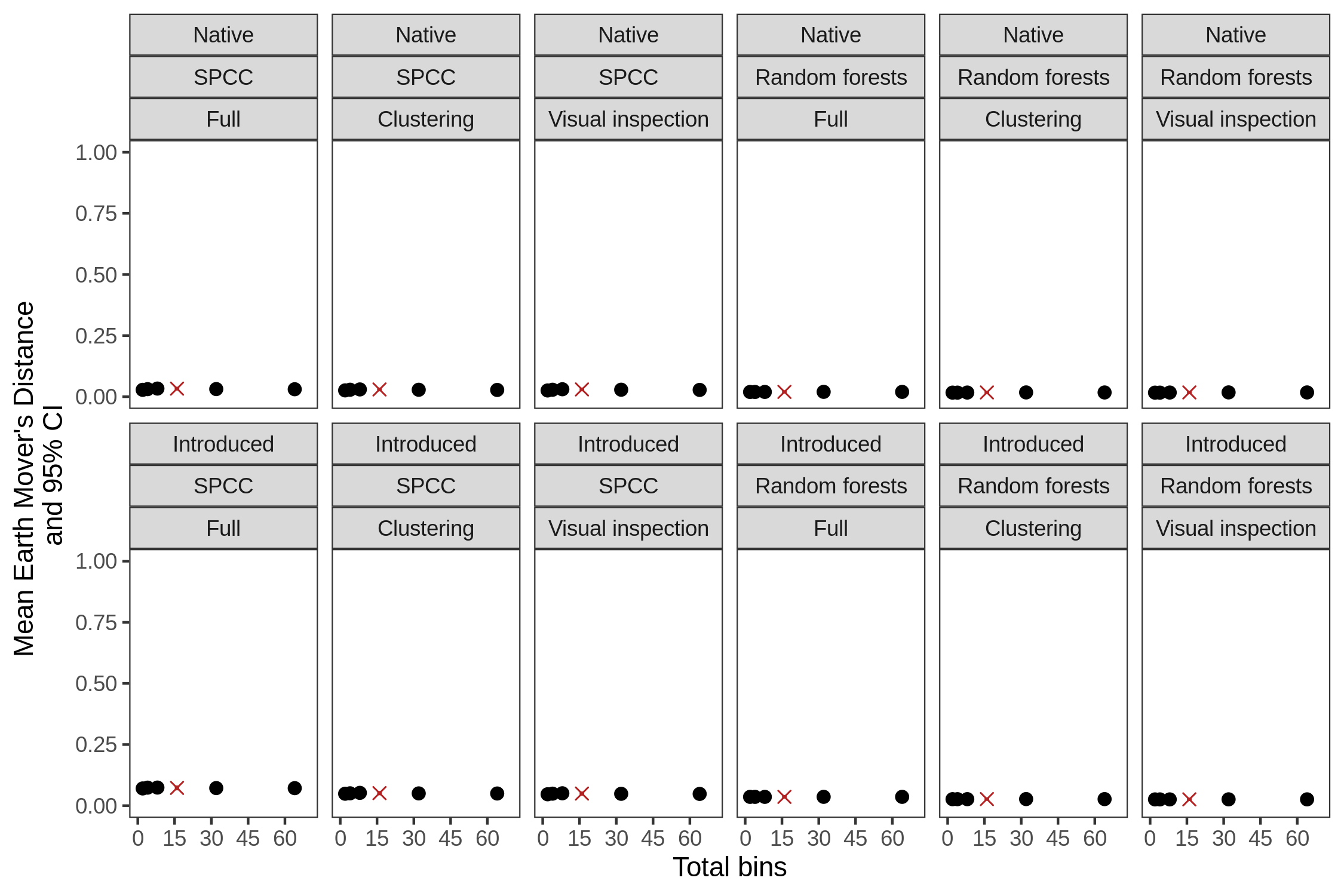

Supplement: S4 Fig — These results were generated using spectrographic cross-correlation and random forests similarity, as well as the three site scale datasets used to address repeated sampling of unmarked individuals. The means and 95% confidence intervals (CIs) were obtained by summarizing across 100 resampling iterations for each bin number. The calculation used to report results in the main text (16 bins) is shown as a red “X”. The 95% CIs are small and are not visible around the mean. (TIFF) [file pcbi.1011231.s005.tiff]

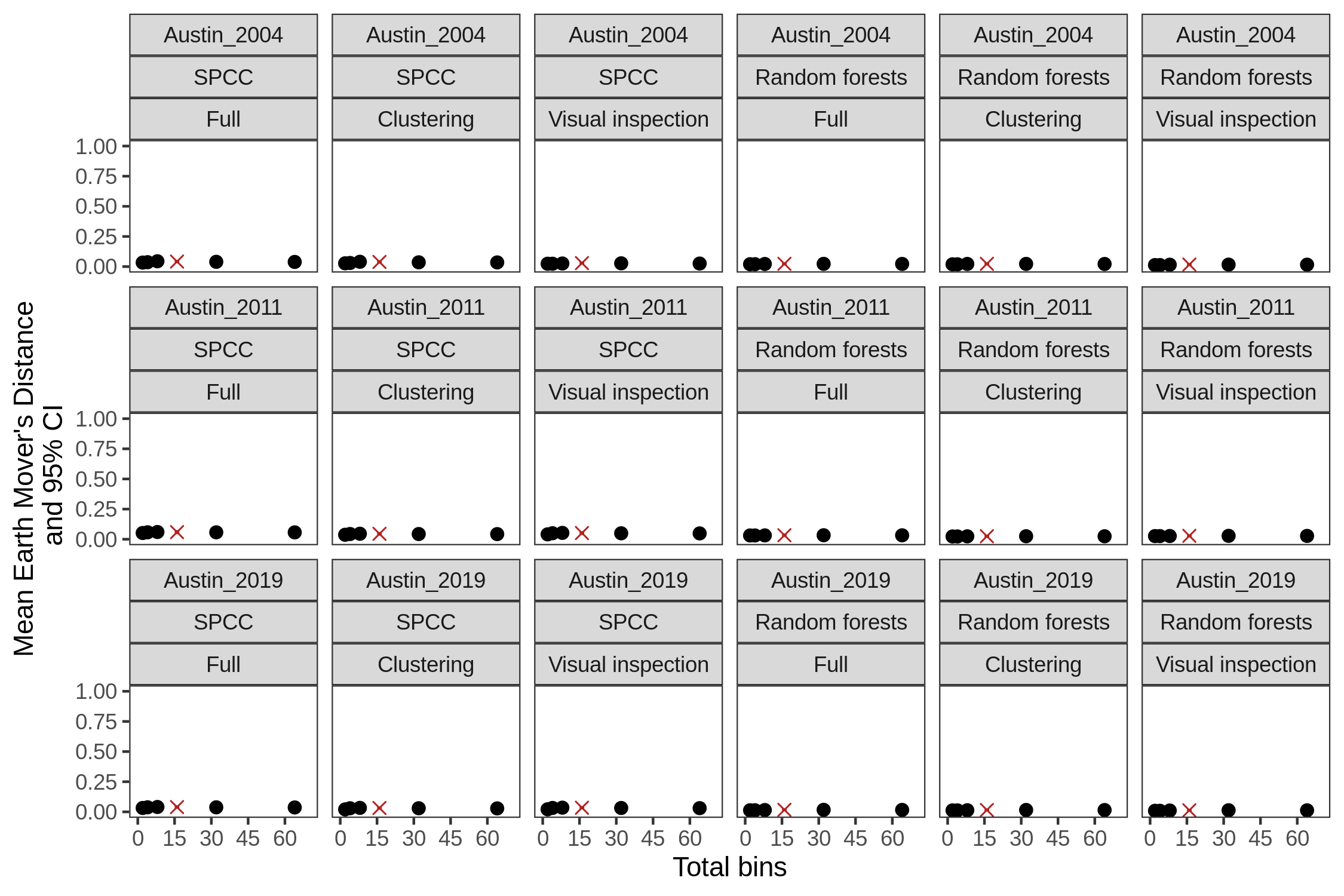

Supplement: S5 Fig — These results were generated using spectrographic cross-correlation and random forests similarity, as well as the three site scale datasets used to address repeated sampling of unmarked individuals. The means and 95% confidence intervals (CIs) were obtained by summarizing across 100 resampling iterations for each bin number. The calculation used to report results in the main text (with 16 bins) is shown as a red “X”. These 95% CIs are also small and are not visible around the mean. (TIFF) [file pcbi.1011231.s006.tiff]

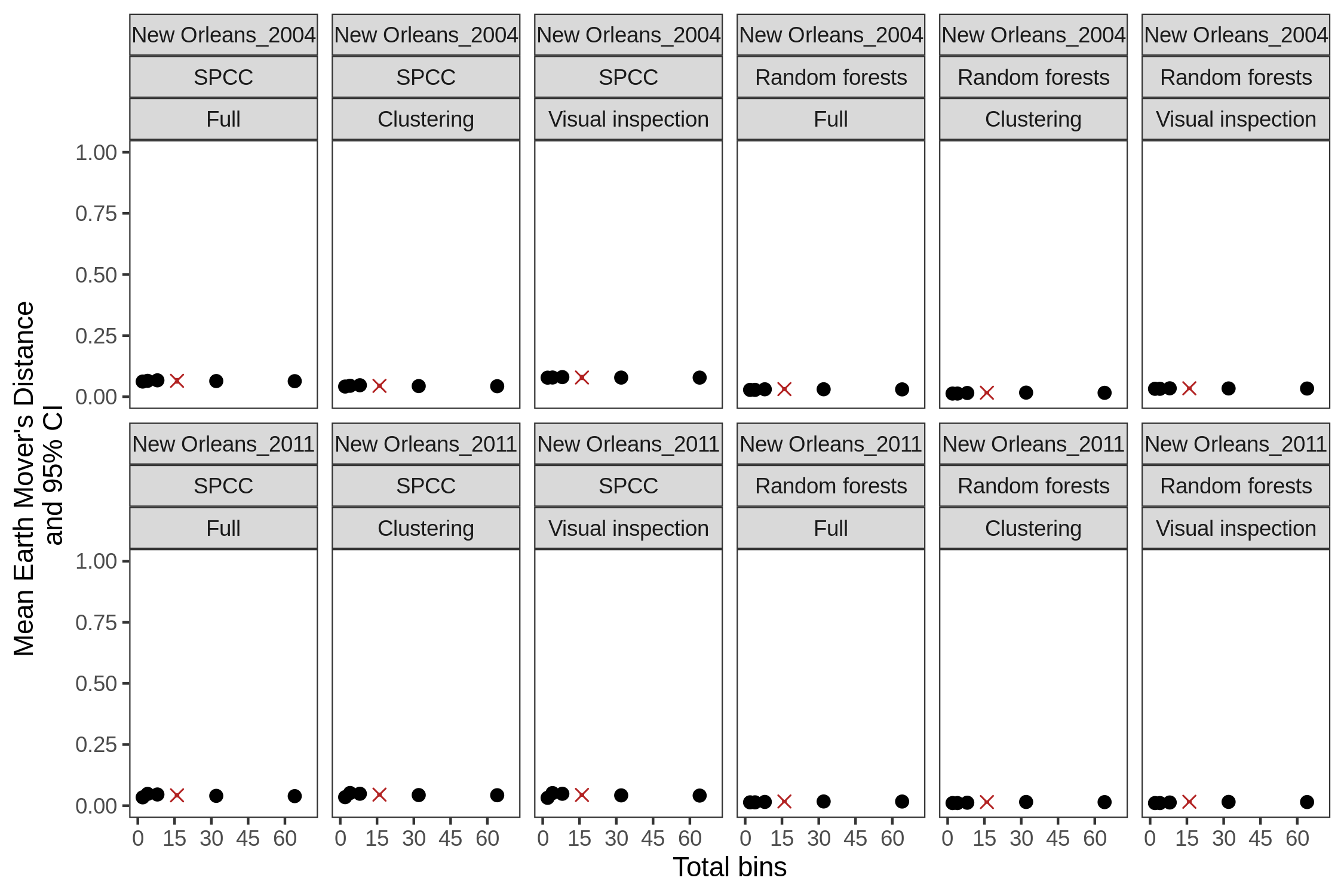

Supplement: S6 Fig — These results were generated using spectrographic cross-correlation and random forests similarity, as well as the three site scale datasets used to address repeated sampling of unmarked individuals. The means and 95% confidence intervals (CIs) were obtained by summarizing across 100 resampling iterations for each bin number. As above, the calculation used to report results in the main text (with 16 bins) is shown as a red “X”, and the 95% CIs are not visible around the mean. (TIFF) [file pcbi.1011231.s007.tiff]

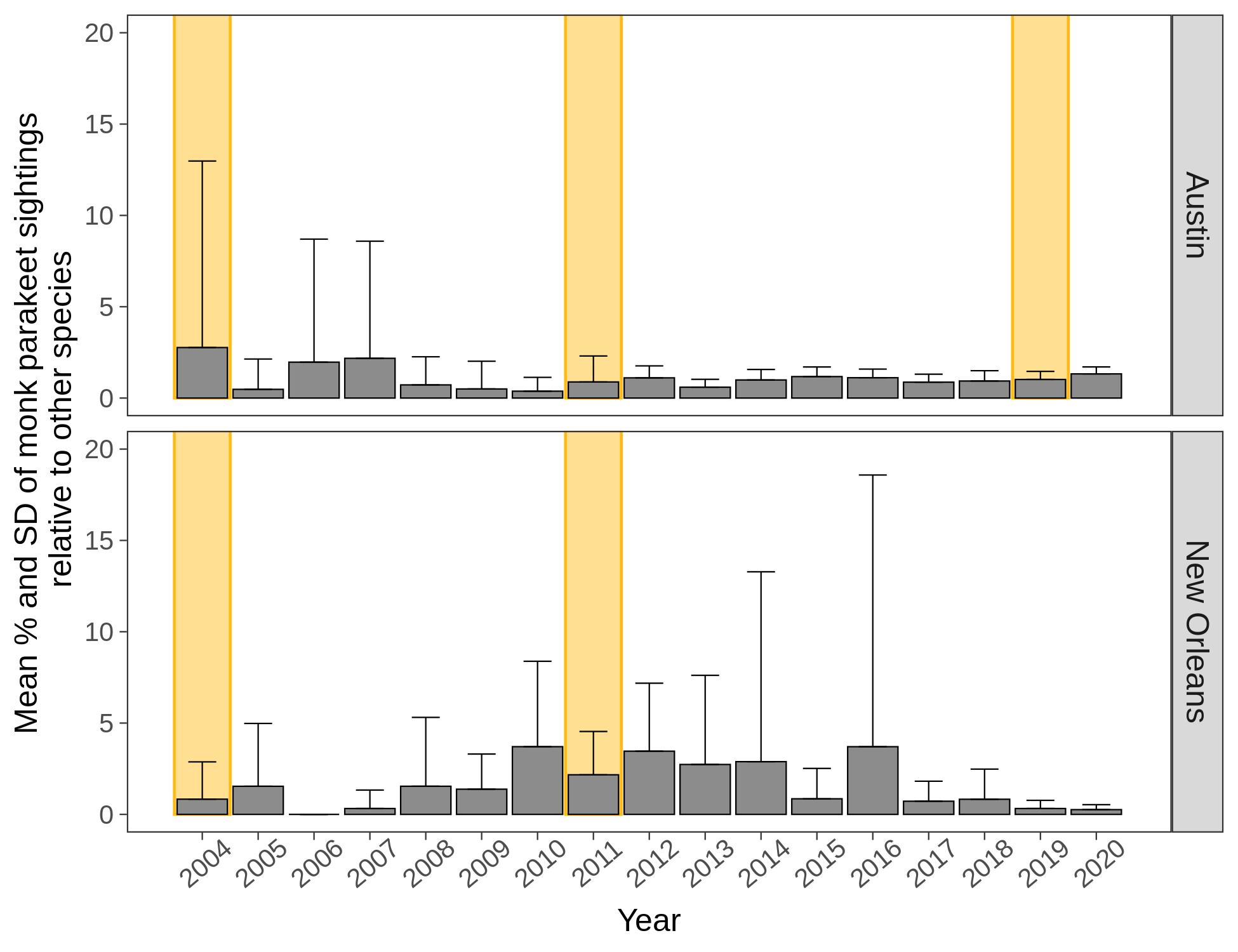

Supplement: S7 Fig — Each bar represents the mean percentage of monk parakeets observed relative to other species, averaged across weeks per year. The error bars denote the standard error. Gold rectangles highlight the sampling years in which monk parakeets were recorded in each city. (JPEG) [file pcbi.1011231.s008.jpeg]

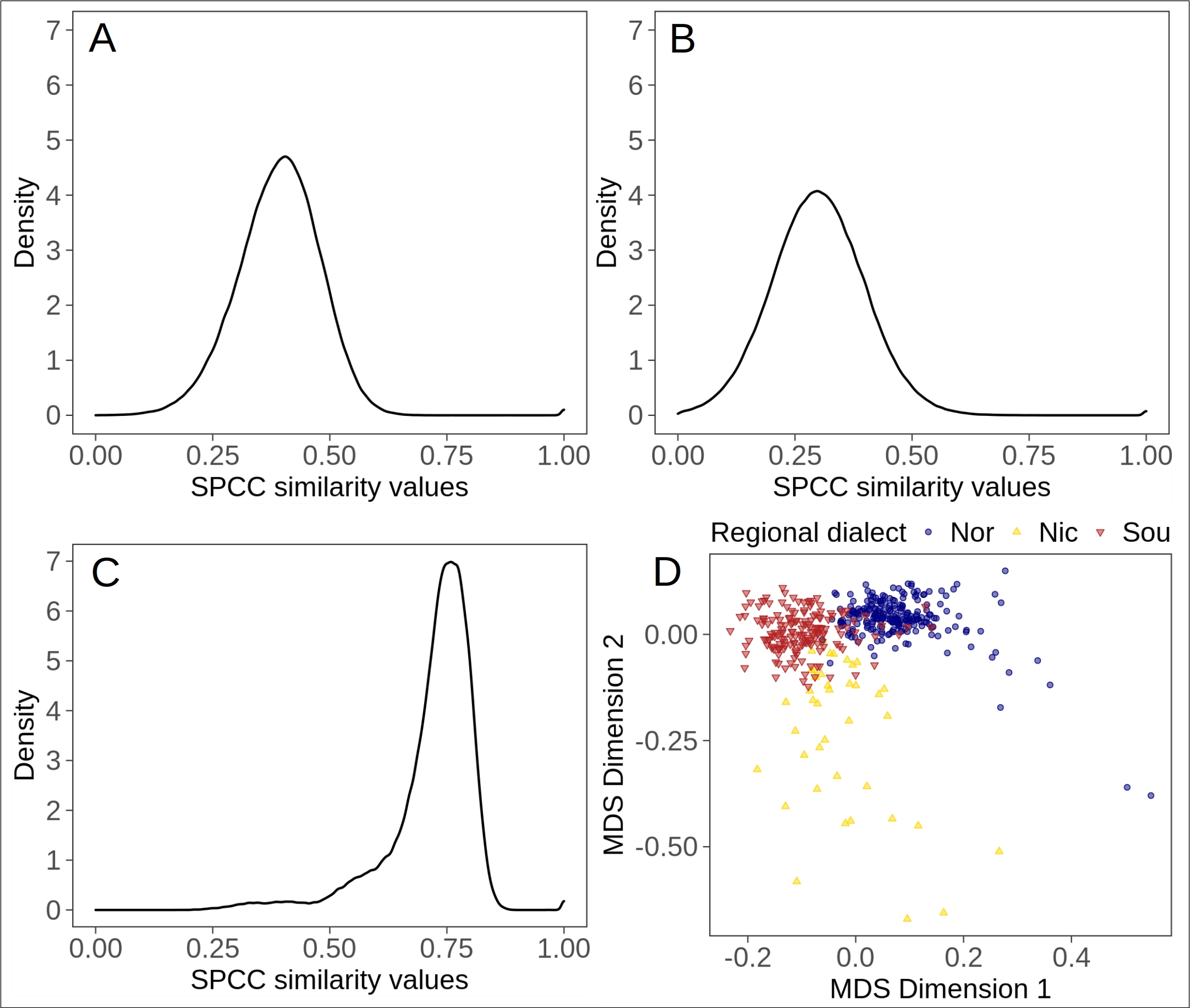

Supplement: S8 Fig — Panels A, B, and C show density curves of SPCC values for native range monk parakeets, introduced range monk parakeets, and yellow-naped amazons, respectively. Each density curve was generated from the full symmetric matrix of similarity values for the given species and range (including the diagonal). Panel D shows acoustic space for yellow-naped amazon contact calls, and points are colored by three regional dialects reported in Costa Rica by [23] (Nor = North, Nic = Nicaragua, Sou = South). We used these graphics to doublecheck the similarity values that we used for our comparative analysis. (JPEG) [file pcbi.1011231.s009.jpeg]
